# Ultrafast solvent migration in an iron complex revealed by nonadiabatic dynamics simulations†

**DOI:** 10.1039/d5sc01174d

**Published:** 2025-05-13

**Authors:** Severin Polonius, Leticia González, Sebastian Mai

**Affiliations:** a Institute of Theoretical Chemistry, University of Vienna Währinger Straße 17 1090 Vienna Austria; b University of Vienna, Vienna Doctoral School in Chemistry (DoSChem) Währinger Str. 42 1090 Vienna Austria; c Research Platform on Accelerating Photoreaction Discovery (ViRAPID), University of Vienna Währinger Strasse 17 1090 Vienna Austria leticia.gonzalez@univie.ac.at sebastian.mai@univie.ac.at

## Abstract

The response of a solvation shell to molecular solute photoexcitation is an ubiquitous phenomenon of great relevance in chemistry. This response can occur within just few tens of femtoseconds, making it very challenging to resolve experimentally. Thus, the details of the (an)isotropy of the solvent response around a solute, the presence of coherent solvent fluctuations, hydrogen bond reorganization mechanisms, and the intricate interplay between electronic, spin, nuclear, and solvent dynamics remain elusive. Here, we report large-scale nonadiabatic molecular dynamics simulations of [Fe(CN)_4_(bipy)]^2−^ (bipy=2,2′-bipyridine) in water, where the electronic evolution from singlet metal-to-ligand charge transfer (MLCT) states to triplet MLCT and metal-centered (MC) states overlaps temporally with the molecule's nuclear motion and a strong solvent shell response. We leverage vibronic coupling model potentials combined with electrostatic embedding, within our so-called vibronic coupling/molecular mechanics (VC/MM) method, to be able to compute several thousand nonadiabatic excited-state trajectories, including all relevant singlet and triplet states as well as several thousand explicit water molecules. This superior statistics affords an unprecedented view on the three-dimensional solvent distribution dynamics at few-fs and sub-Å resolution. The results reveal a direct solvent migration mechanism, where excitation to the MLCT states leads to the breaking of hydrogen bonds to the cyanide ligands within less than 100 fs, followed by the formation of hydrogen bonds with the negatively charged bipyridyl ligand by the same water molecules. Furthermore, the MLCT and MC states show very distinct solvent responses, which are overlapping in time, as governed by the electronic dynamics. More broadly, this work demonstrates how VC/MM nonadiabatic dynamics simulations can resolve anisotropic solvent dynamics around a photoexcited solute with unprecedented detail, offering a new perspective that could stimulate the development of time-resolved experimental techniques capable of probing such solvent behaviour.

## Abstract 2

Large scale surface hopping simulations of [Fe(CN)_4_(bipy)]^2−^ in water elucidate the photoinduced dynamics of the solvation shell. The ultrafast reorganization and migration of water molecules is primarily driven by changes in the electronic state.

## Graphical abstract

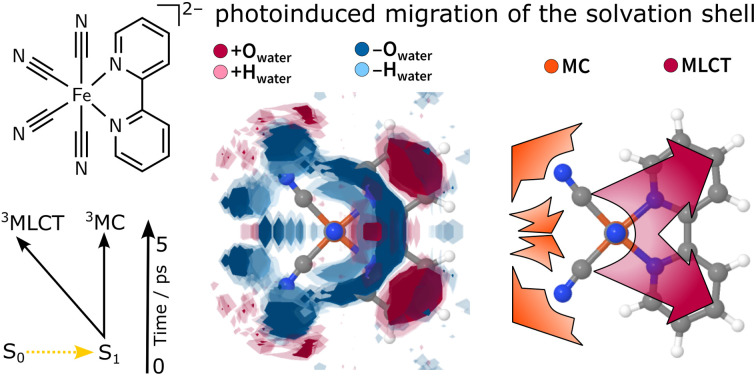

## 1 Introduction

Understanding the interaction of a solute with the surrounding solvent molecules is essential to control reactivity in chemistry.^1–4^ When reactions are initiated by light, the dynamics can be studied by a number of ultrafast experimental techniques that provide information on the solute–solvent interactions over time. The evolution of the solute's electronic wave function can be inferred from methods such as transient absorption spectroscopy,^5–7^ time-resolved X-ray fluorescence^8–10^ or photoelectron spectroscopy.^11,12^ The solute's vibrational response can be investigated through ultrafast X-ray scattering experiments,^7,8,13–18^ which are also able to measure the solvent response occurring through libration and diffusion.^3,19^ Yet, obtaining time-resolved information on specific or strongly localized intermolecular interactions (such as hydrogen bonding), which influence both the nuclear and electronic solute properties on very short time scales, is particularly challenging.

Hydrogen bonds play a significant role in modulating photochemistry because they alter the electronic structure of the solute, stabilizing or destabilizing particular electronic states.^2,20–23^ This, in turn, can shift absorption spectra, induce specific conformational changes, or impact deactivation mechanisms, reaction rates, and excited-state lifetimes. Transition metal complexes can be particularly amenable to changes in reactivity through solvent interactions.^24,25^ One example is [Fe(CN)_4_(bipy)]^2−^, which experiences a particularly strong ligand field introduced by the cyanide and bipyridyl ligands,^26–28^ which, combined with its high charge and polarity, destabilizes the metal-centered (MC) states in favor of the metal-to-ligand charge transfer (MLCT) ones.^29^ Compared to 4d/5d metal analogues, Earth-abundant transition metal complexes based on 3d metals like Fe tend to have short MLCT lifetimes, limiting their use as photocatalysts.^30,31^ Interestingly, the solvent has a considerable effect on the excited-state dynamics of [Fe(CN)_4_(bipy)]^2−^: while in water the MLCT states are quenched in less than 200 fs,^9,29^ in dimethyl sulfoxide the lifetimes extend to tens of picoseconds.^32,33^

A recent nonadiabatic dynamics study of [Fe(CN)_4_(bipy)]^2−^ in explicit water^34^ revealed that solvent reorganization occurs on an ultrafast timescale, with water rearranging in about 50 fs around the cyanides, and more slowly around the bpy ligand, while the electronic and Fe–ligand bond dynamics take place over longer timescales. Given the very high computational cost associated with performing *ab initio* excited-state simulations on transition metal complexes,^35^ and even more so in explicit solution, it has not been possible to resolve in detail the time evolution of the solute–solvent interactions so far. Within the widely used surface hopping methodology,^36^ investigating non-equilibrium solvent–solute dynamics in three dimensions requires an extremely large amount of trajectories,^37^ which is computationally unfeasible for on-the-fly simulations.

Here, we apply our recently developed hybrid vibronic coupling model embedded into a molecular mechanics environment (VC/MM)^38^ method to characterize the solvent dynamics around [Fe(CN)_4_(bipy)]^2−^*via* time-dependent three-dimensional spatial distribution functions (TD-3D-SDFs).^37^ The several thousand surface hopping trajectories afforded through this approach provide an unprecedented spatially resolved view of the photoinduced dynamical changes within the solvation shell over time, allowing one to determine whether solvent reorganization is homogeneous or inhomogeneous around the molecule, and how strongly the solvent response is damped. Additionally, it enables us to investigate whether the solvation shell evolves *via* directed migration of hydrogen-bonded waters or through a bulk-exchange mechanism, as well as to reveal correlations in the solvation structure with the electronic charge transfer character. Understanding the interaction between [Fe(CN)_4_(bipy)]^2−^ and its solvent, especially its impact on the MC states, is essential to advance the design of more efficient 3d metal-based complexes for photocatalytic applications. Furthermore, our work discusses how inhomogeneous solvent distribution dynamics around the solute could be experimentally detected, to stimulate novel experimental techniques that can provide access to the three-dimensional solvent distributions.

## 2 Methods

The excited state dynamics of [Fe(CN)_4_(bipy)]^2−^ is carried out using trajectory surface hopping simulations using SHARC^39,40^ and a vibronic coupling/molecular mechanics (VC/MM) model^38^ including all linear and a number of selected quadratic coupling terms. The VC model has been parameterized^41,42^ in a diabatic basis of 21 singlet and 20 triplet states with time-dependent density functional theory (TDDFT), where energies and gradients were obtained from GAUSSIAN16 (ref. 43) and spin–orbit couplings from ORCA5.^44^ We employed the B3LYP* functional,^45^ using the def2-TZVP (for Fe) and def2-SVP all-electron basis sets (for other atoms), the GD3BJ empirical dispersion correction,^44,46^ and the Tamm–Dancoff approximation.^47^ The charge transfer character is computed with TheoDORE,^48^ where—due to strong bonding—we consider^49^ the Fe(CN)_4_ unit as the metal (M) and the bipy unit as the ligand (L). Further simulation details can be found in Sections S1.1 and S1.2 of the ESI.†

Due to the high charge and polarity of [Fe(CN)_4_(bipy)]^2−^, gas-phase calculations have significant convergence problems. However, parametrizing the VC/MM model with implicit solvation would lead to double-counting of solvent shifts once the explicit solvent molecules are included. Hence, the VC parameters were generated using implicit solvation with a dielectric constant set to *ε*_r_ = 1.77 (*i.e.*, the square of the refractive index of water), such that the VC parameters take into account the polarizability of water solvent molecules due to their electron densities. The remaining polarizability of water is accounted for by the motion of the explicit water molecules within the VC/MM simulations (Section S1.1 and Fig. S1 and S2†). Further, we include a selected subset of quadratic vibronic coupling parameters, *i.e.*, state-specific frequency shifts in the form of *γ*^(ii)^_kk_ parameters (Section S1.2 and Fig. S3†) of normal modes that affect the equatorial Fe–ligand distances for all ^3^MC states. Extending the VC model with these *γ*^(ii)^_kk_ parameters significantly improved the accuracy of the MC states in preliminary gas-phase simulations. Both aspects, including implicit solvation into the parameters and including quadratic parameters, go beyond previous LVC/MM simulations.^37,38^

In order to obtain enough trajectories to resolve the 3D solvation structure, we generated 30 000 initial conditions of [Fe(CN)_4_(bipy)]^2−^ in water with classical molecular dynamics simulations that used a flexible water model, as described elsewhere^34,50^ (Section S1.3†). Out of the 30 000 molecular dynamics snapshots, 4473 initial conditions were stochastically selected^51^ in a window between 487 nm to 497 nm (around 2.52 eV) centered on the peak of the first absorption band (Fig. S4†). From them, 4366 initial conditions (97.6%) were excited into the adiabatic S_3_ state, which has MLCT character. All 4473 initial conditions were propagated for 5000 fs on the lowest-lying six singlet and seven triplet adiabatic electronic states, which are enough for describing the excited state dynamics of the system. Details on the surface hopping settings can be found in Section S1.4.†

The analysis of the solvation dynamics is done through TD-3D-SDFs evaluated from a molecular perspective,^37^ where the solute coordinates are aligned to a reference structure at every time step. While previously,^37^ TD-3D-SDFs were constructed as simple 3D histograms, in this work we employed a kernel density estimation,^52,53^ which, although computationally more costly, provides smoother distributions compared to histograms for the same number of data points. The density estimation is based on a Gaussian kernel:1
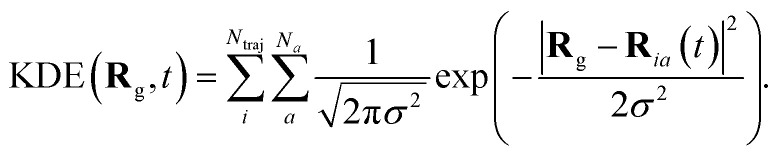
Here, the value of the 3D-SDF at grid point **R**_g_ and time *t* is the sum of 3D Gaussian functions for all *N*_traj_ trajectories and all *N*_*a*_ atoms of the chosen type in the system with coordinates **R**_*ia*_ at time *t*. The variance of the Gaussian function *σ* is set to 0.5 Å, corresponding to the bin width chosen in previous work.^37^ The kernel-density-estimated 3D-SDFs were evaluated on a grid of 40 points in each Cartesian direction and the same 0.5 Å spacing. The 3D-SDFs were symmetrized to the *C*_2v_ point group of [Fe(CN)_4_(bipy)]^2−^ to effectively quadruple the number of sampling points, leading to even better statistics and less noisy distributions. Additionally, we constructed TD-3D-SDFs from subsets of trajectories that exhibit either pure MLCT or MC wave function character to identify the solvent response to the evolving electronic wave function.

## 3 Results and discussion

The dynamical simulations on [Fe(CN)_4_(bipy)]^2−^ are targeted to investigate the electronic, spin, and, particularly, the solvent relaxation dynamics, all of which are intimately coupled with each other. These aspects are discussed in order below.

### 3.1 Electronic population dynamics

**Fig. 1:**
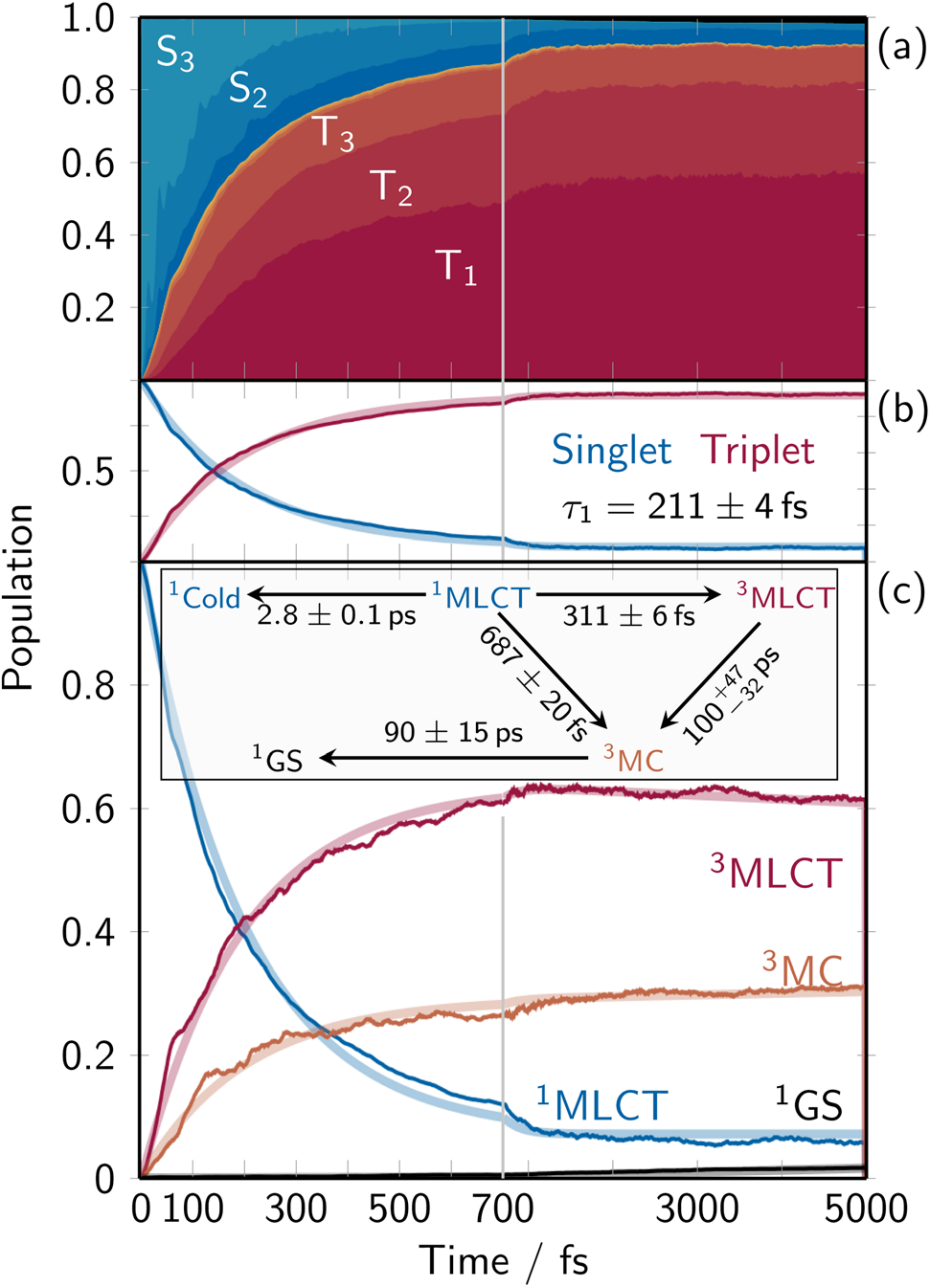
Time-resolved electronic population dynamics. (a) Stacked-area plot showing the contributions from each adiabatic excited state with the singlet ground state (black, top), excited singlet states (dark blue to light blue), and excited triplet states (dark red to light orange). (b) Total singlet and triplet populations (thin lines) and corresponding mono-exponential fits (thick semitransparent lines). (c) Time-dependent diabatic populations (thin lines) for the ground state (GS, black), the ^1^MLCT states that was excited into (blue), the ^3^MC states (orange), and the ^3^MLCT states comprising all other triplet states (majorly MLCT character, red). The thick lines indicate the global fitting results from the shown kinetic model.

As the evolution of the electronic structure is ultimately responsible for the changes undergone in the solvent, we will first analyze the electronic population dynamics after photoexcitation, to understand which states are populated during the relaxation process. Fig. 1a shows the time-resolved adiabatic populations of the considered electronic states. Initially, the singlet population is dominated by the S_3_ state. As the overall singlet population decreases, the proportions gradually shift, with the S_2_ and then S_1_ becoming relatively more prominent due to nonradiative decay. Efficient intersystem crossing (ISC) occurs within the first 1000 fs, resulting in about 90% of the population moving to the triplet manifold, distributed among the three lowest triplet states. At the end of the propagation time, 6% of the population remains in the singlet state (mostly S_1_); this outcome is referred to as “cold” singlet state (of MLCT character) in the kinetic model below. The total population transfer between singlet and triplet states is summarized in Fig. 1b, together with a biexponential fit that takes into account this “cold” singlet. The total singlet population depletes quickly with a time constant *τ* = 211 ± 4 fs from the ^1^MLCT states, and no ^1^MC population is observed. The time constant is in excellent agreement with previous TDDFT-based QM/MM simulations,^34^ which provided a 210 ± 20 fs ISC time constant.

The electronic dynamics in terms of the diabatic populations (defined by the characters of the electronic eigenstates at the ground-state equilibrium geometry) is shown in Fig. 1c, together with a kinetic model. Starting with 100% of the populations in the ^1^MLCT manifold, the electronic population bifurcates *via* ISC into ^3^MC and ^3^MLCT states. The ^3^MC population reaches a final value of about 31% with a trend to increase, while the ^3^MLCT states reach a final population of 62% with a trend to decrease (^3^MLCT life time of 100_−32_^+47^ ps). The singlet ground-state (black line at the very bottom) is very slowly populated with a time constant of 90 ± 15 ps. The bifurcation into the MLCT and MC triplet manifolds occurs with time constants of 311 ± 6 fs and 687 ± 20 fs, respectively, populating the states with a ratio of about 2.2 : 1. The aforementioned “cold” singlet population, labeled as ^1^Cold, is populated slowly with a constant of about 2.8 ps. Accordingly, the final populations at *t* = 5000 fs are 62% ^3^MLCT, 31% ^3^MC, 6% cold ^1^MLCT, and 2% ground state.

The obtained time scales and fitted time constants are not identical to those obtained in previous work,^34^ due to the approximations involved in the parameterization of the VC model (Section S2.1 and Fig. S5†). In particular, while the inclusion of state-specific quadratic coupling terms has shown merit for a better description of the system (compared to preliminary simulations), Fig. S6 and S7† indicate that the ^3^MC states would benefit from an anharmonic description of the Fe–X bond lengths. Consequently, the ^3^MLCT →^3^MC and ^3^MC → ground state dynamics are slower than in TDDFT and experiment. These deviations do not arise from the fact that the fitted time constants are much longer than the simulation time. In fact, the very large number of trajectories affords reasonably small uncertainties, despite the fact that the time constants are much longer than the simulated time (90 respectively 100 ps *versus* 5 ps).

### 3.2 Response of the solvation shell

**Fig. 2:**
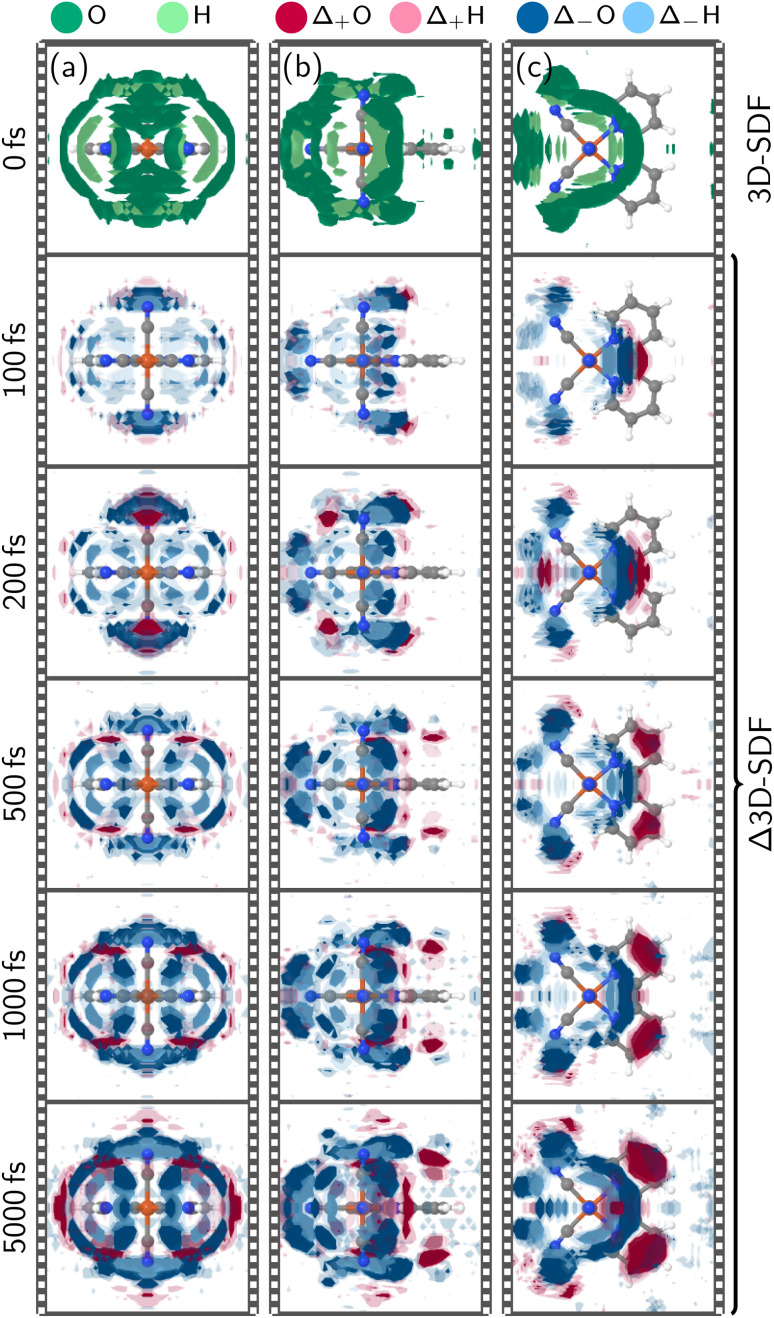
Symmetry-adapted three-dimensional spatial distribution functions (3D-SDFs) of water oxygen and hydrogen atoms at *t* = 0 fs and time-dependent difference 3D-SDFs thereafter. In all panels at *t* = 0 fs, spatial regions with an occurrence higher than 2.5 times the average are colored green for oxygen atoms and light green for hydrogen atoms. For the Δ3D-SDFs at *t* > 0 fs, the iso value is set to 0.5 and 0.3 times the average with solid and shaded colors respectively; positive deviations are colored with red colors and negative deviations, with blue colors with oxygens in the darker and hydrogens in the lighter shade. Panels (a–c) show different orientations of the system.

Next, we focus on the analysis of the structural changes within the solvation shell, which is the core result of the present work. Fig. 2 collects the dynamical response of the solvation shell around [Fe(CN)_4_(bipy)]^2−^ over time from different viewing angles (see Fig. S8 and S9 in Section S2.2† for larger images). At *t* = 0, a strong solvation shell surrounding the cyanide ligands is visible, forming rings around the four nitrogen atoms. The features around the axial cyanide ligands are more extensive and reach above/below the nitrogen atoms on the bipyridyl ligand. As expected from the aromatic, hydrophobic bipyidyl ligand, the 3D-SDFs do not indicate strong hydrogen bonds or other structure in the solvation shell around the bipyridyl carbon atoms. Within 100 fs after excitation, the solvation shell starts to deplete around all nitrogen atoms, particularly in the vicinity of the bipyridyl nitrogen atoms. At the equatorial cyanide ligands, this decrease is only observable on the outward-facing side. At the axial cyanides, the oxygen atom occurrence is visibly shifted towards the C–C bridge of the bipyridyl ligand (Fig. 2c). This trend continues beyond *t* = 200 fs. Around 500 fs, the oxygen feature at the C–C bridge splits and moves to accumulate above the two bipyridyl *para* carbon atoms, forming notable hydrogen bonds to the bipyridyl aromatic system^54^ that is negatively charged in the MLCT state. For later times until *t* = 5000 fs, the developed features—rings of diminished solvent density around the cyanides and the newly formed hydrogen bonds—intensify at a somewhat slower pace.

The solvation shell dynamics as represented by the TD-3D-SDFs provides a much more holistic depiction of the dynamics, compared to the radial distribution functions (RDFs) that are collected in Section S2.3 and Fig. S10–S12.† Nonetheless, the RDFs agree on the finding of a fast inertial solvent response leading to a reduction in hydrogen bonding to the cyanide groups. A monoexponential fit of the first temporal component of the singular value decomposition of the time-dependent RDF provides time constants of 50–75 fs for the initial solvent response. This fully agrees with a 75 fs time constant obtained from the singular value decomposition of the 3D-SDF, and is also consistent with other computed and experimental time constants for such responses.^3,19,34,55^ In the time-dependent RDFs (Fig. S10 and S12†), one can additionally observe several oscillatory features in the distribution of the C_bpy_–H_sol_, C_CN_–H_sol_, or N–H_sol_ distances. Such oscillations are of significant interest because they provide insight into relevant coherent vibrational modes, and, depending on the atoms involved in the vibrations, generally can be observed in X-ray solvent scattering experiments.^34^ Here, based on the 3D-SDFs, we find that for [Fe(CN)_4_(bipy)]^2−^ these oscillations arise almost exclusively from intramolecular solute vibrations that modulate the solute–solvent distances, rather than from coherent fluctuations within the solvent. Making this distinction is only possible with the 3D-SDFs, because it is based on the actual three-dimensional coordinates of all solute and solvent atoms, rather than on inter-atomic distances that can only describe relative motion. We note that Section S2.3† also provides a comparison of the RDFs obtained in the present work with the ones obtained in previous on-the-fly TDDFT/MM simulations (Fig. S11†).^34^ Here, we obtain excellent agreement within the limits of the TDDFT/MM simulations (lower statistics, shorter simulation times), where all extrema and shoulders of the RDFs and their temporal behavior are reproduced in the VC/MM simulations. We conclude from this comparison to the TDDFT/MM data and from the good agreement with experimental solvent response time constants that the overall solvent dynamics of [Fe(CN)_4_(bipy)]^2−^ is accurately captured in our present simulations.

**Fig. 3:**
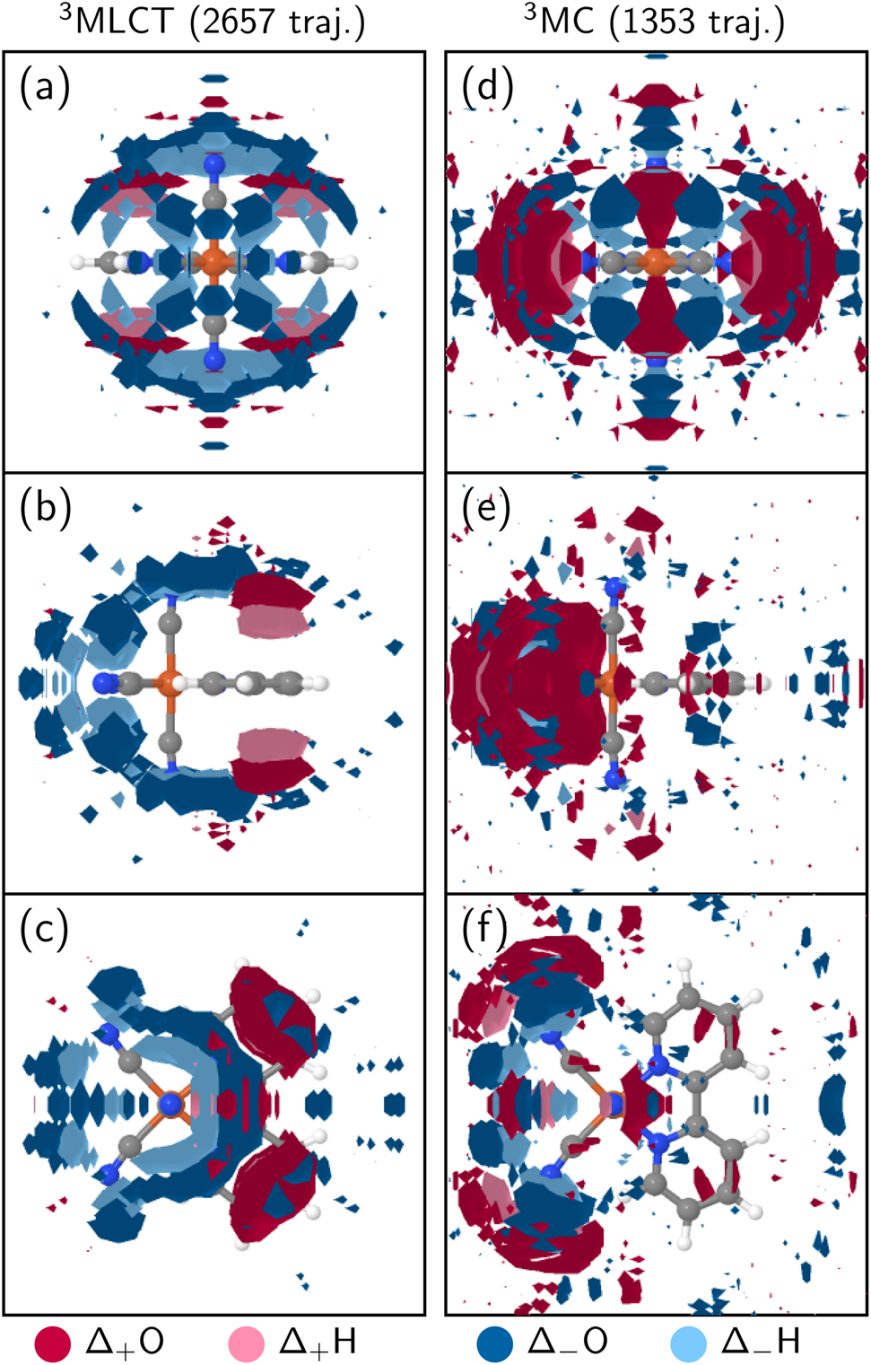
Average Δ3D-SDFs for a subset of trajectories with dominant ^3^MLCT (a–c, 2657 trajectories) and ^3^MC (d–f, 1353 trajectories) character at *t* = 5000 fs (differences relative to the ground-state equilibrium). The used subset of trajectories had a stable population in the respective states of at least 76% for the last 1000 fs. In all panels, the iso value was set to 0.5 times the average; positive deviations are colored with red and negative ones with blue, with oxygens in the darker and hydrogens in the lighter shade.

As shown in Fig. 1, both ^3^MLCT and ^3^MC states are populated throughout the simulation, so Fig. 2 represents a complex superposition of the solvent dynamics around MLCT and MC states and the electronic dynamics. To disentangle the solvent response of these two characters, Fig. 3 visualizes the Δ3D-SDFs at 5000 fs (relative to the ground state equilibrium) of subsets of trajectories that evolve dominantly in pure ^3^MLCT or ^3^MC states, respectively. Both subsets were selected to have at least 90% of either character for 84% of the final 1000 fs. This means that the chosen trajectories had either a ^3^MLCT or ^3^MC population of at least 76% in that time frame. Hence, the shown Δ3D-SDFs are characteristic for the solvent shell of the ^3^MLCT and ^3^MC states, respectively. We note that, due to the smaller number of trajectories, these Δ3D-SDFs are somewhat more noisy^37^ than the ones in Fig. 2, and because the trajectories enter the MLCT/MC state at different times, we only show the distributions at 5000 fs. In the ^3^MLCT manifold (Fig. 3a–c), water recedes from the cyanide ligands and increases almost exclusively above and below the bipy ligand with respect to the ground-state equilibrium (Fig. 2a–c). Especially panel b shows that hydrogens (light red) are closer to the bipyridyl than oxygens (darker red), indicating that the ligand serves as hydrogen bond acceptor in the ^3^MLCT state. This is consistent with the partial charges of the ^3^MLCT diabatic states, shown in Section S2.4 in Fig. S13,† where notably the *para*-carbon atom of the bipyridyl ligand changes from positively to negatively charged from the ground state to the ^3^MLCT states. Fig. 3a–c also shows that the solvation shell decreases more at the axial cyanide ligands than at the equatorial ones. This can be traced to the fact that the lowest ^3^MLCT states involve the out-of-plane t_2g_ orbitals that are delocalized over the axial cyanides.^34^ For the ^3^MC state (panel d–f), no significant changes in the bipyridyl solvent shell with respect to the ground state can be observed, as the MC excitation does not affect this ligand. Instead, a significant rearrangement of the cyanide solvation shell can be observed. Here, the strongest effect is the receding of the solvation shell from the equatorial cyanides, which correlates with the Fe–C bond length changes of the ^3^MC states (Fig. S7†) and the corresponding RDF (Fig. S12†), *i.e.*, the solvent is pushed outwards by the elongating Fe–C bonds.

### 3.3 Characterization of solvent response

Besides the direct description of the temporal evolution of the solvent shell around [Fe(CN)_4_(bipy)]^2−^—as discernible from the 3D-SDFs—our simulations can shed light on a number of additional questions, as posed in the introduction. We begin with “How inhomogeneous is the solvent response around the molecule?”, where, in particular, we refer to the time constants of solvent response around the different moieties. An analysis of the time-dependent hydrogen bond counts (Section S2.5 and Fig. S14†) reveals that the breaking of the hydrogen bonds to the cyanide ligands occurs biexponentially with time constants of about 15 and 240 fs. The new hydrogen bonds to the bipyridyl are formed more slowly, with time constants of 30 and 500 fs. Reasons for this behaviour are the more localized change in charge on the cyanides (compared to the delocalized charge on the bipyridyl), as well as that the waters forming the bipyridyl hydrogen bonds must first be released from the cyanides, as we will discuss in more detail below. The different time scales of breaking and forming of hydrogen bonds can also be extracted from the time-dependent RDFs (Section S2.3 and Fig. S10 and S12†). Quantifying the solvent response near the cyanide and bipyridine ligands provides time constants of 50–75 fs and 280 fs, respectively. Moreover, the obtained high-fidelity RDFs afford to identify the differences in the solvent dynamics around the equatorial and axial cyanide ligands (Fig. S12†), an endeavor that was not feasible before.^34^ We find that the axial cyanides show a stronger response in their first solvation shell compared to the equatorial ones. Interestingly, both kinds of cyanide solvent shells show some oscillatory response in the RDFs, but with different oscillation periods of 80 fs (axial) and 160 fs (equatorial). All these results support the finding that the solvent dynamics is very inhomogeneous around the molecule.

The second major question posed in the introduction can be phrased as “How is the general solvent behavior and how strong is the solvent response damped?”. Here, the RDFs in Fig. S10 and S12† and the ones in ref. 34 (Fig. S11†) exhibit significant coherent oscillations, which can potentially be observed in time-dependent X-ray scattering experiments if signal strength permits. Such coherent oscillations in the RDFs could be taken as indications of a relatively weakly damped (*i.e.*, underdamped) solvent motion, where the solvent molecules oscillate back and forth around the new equilibrium positions governed by the excited solute. In order to verify whether such underdamped solvent dynamics actually takes place, we have investigated various slices through the 3D-SDFs shown in Fig. 2. As shown in selected slices in Section S2.6 (Fig. S15 and S16),† no notable oscillatory dynamics of the solvent molecules themselves are present. Instead, the solvent relaxes in a relatively strongly damped fashion, due to the large degree of disorder and the many interactions among the solvent molecules. The oscillations in Fig. S10 and S12† can thus be assigned to coherent oscillations in the solute molecule, *i.e.*, to coherent vibrations of solute molecules relative to less mobile solvent molecules. This finding is fully consistent with recent X-ray solvent scattering experiments on photoexcited iodide ions,^56^ where no oscillatory solvent response was measured due to the absence of internal degrees of freedom in the (monoatomic) solute.

**Fig. 4:**
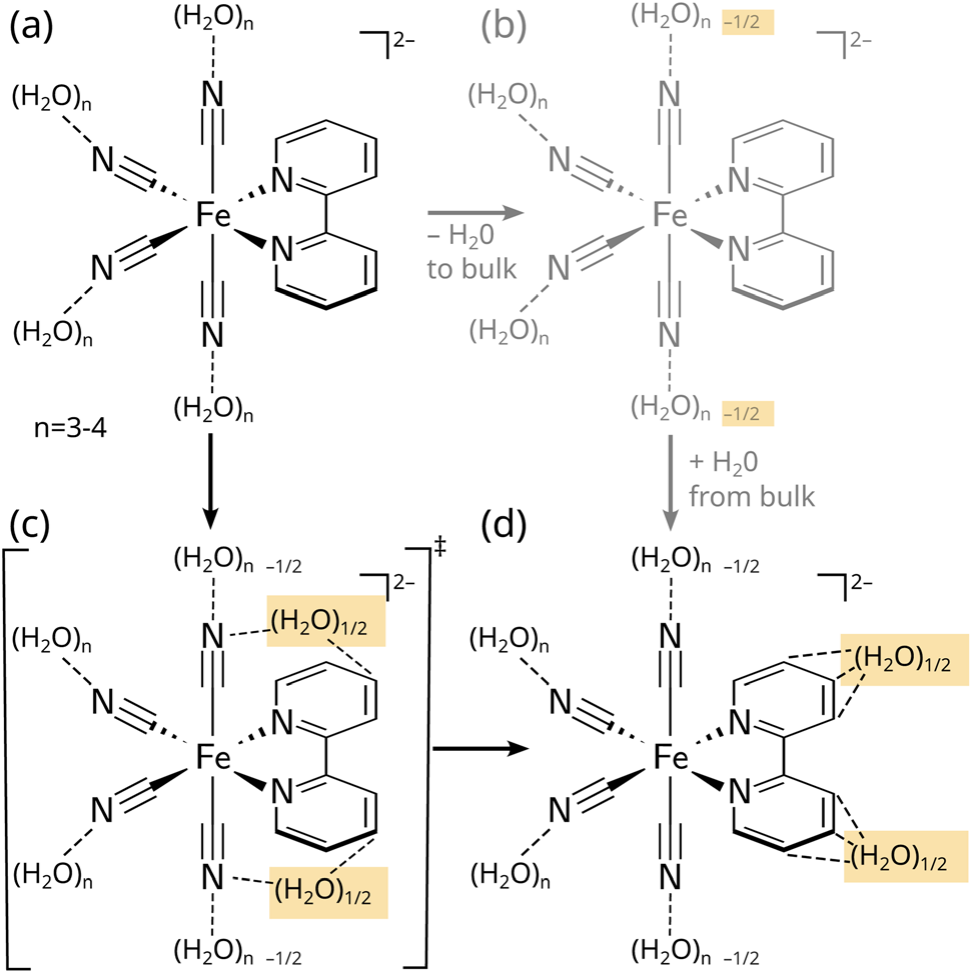
Schematic representation of the initial hydrogen bonding state of [Fe(CN)_4_(bipy)]^2−^ in the ground state (a)—with three to four hydrogen bonds per cyanide—and the rearrangement (b and c) and final hydrogen bonding state (d) in the ^3^MLCT states. The upper right (b) pathway shows a hypothetical bulk exchange mechanism, whereas the lower left (c) pathway illustrates the direct migration pathway found for [Fe(CN)_4_(bipy)]^2−^, as discussed in the text.

The third question can be stated as “What is the mechanism of the solvent response and the breaking and forming of hydrogen bonds?”. Here, we are primarily interested in a statistically meaningful answer about where each solvent molecule is located at *t* = 0 and where it is located at the end of the simulation time. In Section S2.6 (Fig. S17 and Table S2),† we have performed such an analysis, by counting the number of water molecules starting and finishing in several volume regions. The most relevant regions are the first solvation shells of the axial and equatorial cyanides and of the bipyridyl ligand (3 regions), the second solvation shells of the cyanides and the bipyridyl (2 regions), and the bulk (see Fig. S17†). Table S2† provides the corresponding correlation matrix after the full 5000 fs simulation time. Here, the most important entries correspond to the amount of water molecules originating and ending in the first solvation shell. Note that the number of water molecules in each region does not change strongly due to the incompressibility of water. Nonetheless, we find that the first solvation shell loses approximately one water and the second solvation shell gains about that much, which is due to the general receding of the water from the excited solvent and also from the fact that the solute expands slightly in the ^3^MC states. As illustrated in Fig. 4, the analysis shows that waters lost from equatorial cyanides move to the cyanide second solvation shell (in the case of ^3^MC states), whereas waters lost from axial cyanides end up coordinating the bipyridyl (in the case of ^3^MLCT states). In contrast, no extensive exchange of waters between the first solvation shell and the bulk is observed. This shows that hydrogen bond reorganization in [Fe(CN)_4_(bipy)]^2−^ is directed (Fig. 4a → c → d)—the water molecules detached from the axial cyanides and the water molecules attached to the bipyridine ligand are in fact the same molecules that migrate along the surface of the solute cavity to form the new hydrogen bonds. This direct mechanism is in contrast to a (more trivial) bulk exchange mechanism (Fig. 4a → b → d), where detached water molecules would leave the molecule and different water molecules from the bulk would form the new hydrogen bonds.

### 3.4 Experimental observability

As the last part of the discussion, we want to comment on the feasibility of experimentally observing the three-dimensional solvent relaxation dynamics around [Fe(CN)_4_(bipy)]^2−^. This aspect is important to verify our simulations, but we also want to provide a stimulus for future experiments that go beyond the current state of the art in observing anisotropic solvent dynamics.

The fundamental problem in detecting the three-dimensional solvent relaxation dynamics is the fact that, in solution, the solute is randomly oriented and therefore the X-ray scattering signal^13^ is isotropically averaged. Hence, the X-ray scattering signal does not contain anisotropic—or three-dimensional—structural information. However, if the solute molecules can be aligned to some extent, then anisotropic information can be recovered. The most common and simplest method to achieve this is by using a linearly polarized pump laser, which for many solute molecules will produce an ensemble of excited molecules that are preferentially oriented with their transition dipole moment along the pump polarization axis. Depending on the angle between pump polarization vector and X-ray propagation vector,^57,58^ different scattering signals are then obtained that contain information about distribution functions in different directions relative to the polarization vector.^59–61^

In order to assess to which extent the inhomogeneous/anisotropic solvent dynamics around [Fe(CN)_4_(bipy)]^2−^ (as discussed in Sections 3.2 and 3.3) is reflected in anisotropic X-ray scattering experiments, we have computed time-dependent difference RDFs weighted by the Cartesian direction of the distance vectors. From these, we have computed approximate X-ray scattering signals that are also resolved by Cartesian direction. All details can be found in Section S2.8 of the ESI.† Fig. S18 to S21† present the time-dependent difference RDFs for all solute–solvent atom pairs including their Cartesian components. We find that the most significant directionality can be observed for the solute C atoms. Here, one obtains a strong increase of water density at 2–3 Å in the direction orthogonal to the bipyridine ligand's molecular plane (*X* in Section S2.8†), originating from the formation of hydrogen bonds above/below the bipyridine in the MLCT states. Very different distributions are obtained in the two other directions (*Y*: along the bipyridine's long axis; *Z*: along the molecule's symmetry axis). For Fe, N, and H atoms, the time-dependent difference RDFs are not strongly dependent on the Cartesian direction, mostly because the hydrogen bonds towards the cyanide ligands encase the molecule in all directions (see Fig. 2 top).

**Fig. 5:**
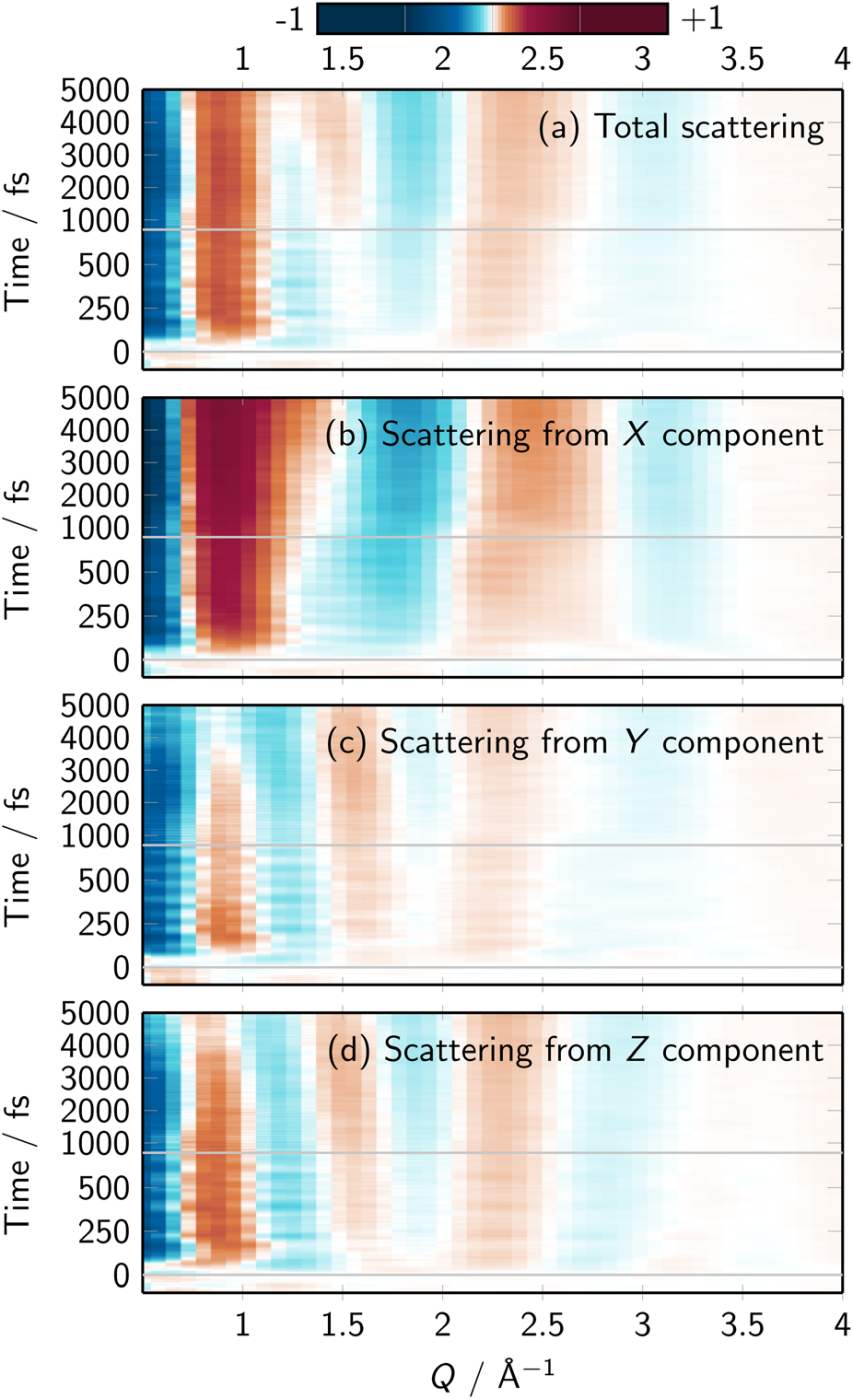
(a–d) Simulated total time-resolved difference scattering signals and its Cartesian components, computed from all solute–solvent RDFs. Details are provided in Section S2.8.† Note that *X* is orthogonal to the bipyridine plane, *Y* is along the bipyridine long axis, *Z* is along the molecule's axis of symmetry. The sum of the *X*, *Y*, and *Z* components is identical to the total signal, although in the figure the total signal was multiplied by 1/3 to enable the usage of the same color scale.

Fig. S22 to S25† present the contributions to the X-ray scattering for different solute elements and Cartesian directions. Just like in the time-dependent difference RDFs, we find a strong directionality in the scattering due to the solute C atoms. Interestingly, this directionality is also found in the summed up X-ray scattering signal, as shown in Fig. 5 (see Fig. S26† for an extended version; the figure corresponds to the solute–solvent cross term, as discussed in the literature^34,62^). The dominance of the C atoms' signal might seem counterintuitive, as Fe is the heaviest element and thus should dominate the scattering signals. However, X-ray scattering intensity is proportional to both the atomic numbers and the number of atoms. In [Fe(CN)_4_(bipy)]^2−^, the sum of atomic numbers of the Fe(CN)_4_ moiety and the one of the bipyridine (C_10_N_2_H_8_) is nearly the same (78 *vs.* 82). Consequently, the scattering due to the (relatively isotropic) distribution around Fe(CN)_4_ and the (very anisotropic) distribution around the bipyridine ligand contribute equally. Overall, Fig. 5 shows that the anisotropic solvent dynamics around [Fe(CN)_4_(bipy)]^2−^ can be followed by means of polarization-dependent X-ray scattering.

It should be mentioned that the polarization-dependent technique described above^59,60^ is primarily sensitive to the difference in solvent distribution parallel and perpendicular to the transition dipole moment. For previous experiments and simulations on this molecule,^29,34^ the molecule was excited into the S_3_ (^1^MLCT) state that is polarized along the molecular symmetry axis (*z*). To observe the accumulation of water above/below the bipyridine ligand, as presented in this work, ideally the molecule would need to be pumped to a bright state with out-of-bipyridine-plane polarization. Alternatively, the molecules would need to be aligned by other techniques, *e.g.*, on surface films.^63^ In a more general sense, additional (indirect) information about the evolution of hydrogen bonds and the state of the solvent could also be obtained through various frequency-domain spectroscopies, *e.g.*, time-resolved infrared, THz, or X-ray absorption spectroscopies and their two-dimensional variants.^64–67^

## 4 Conclusions

Through large-scale, statistically highly robust surface hopping simulations with a vibronic coupling model including electrostatic embedding, we have gained unprecedented insight into the ultrafast solvent response around photoexcited [Fe(CN)_4_(bipy)]^2−^ and how anisotropic X-ray scattering signals can report about it. The solvent relaxation mechanism can be summarized as follows. In the electronic ground state, water forms rings of hydrogen bonds around each of the four negatively charged cyanide ligands, and only a very weak solvation shell around the bipyridyl ligand. After excitation to a bright ^1^MLCT state, water recedes from the axial and equatorial cyanides with 50–75 fs time constants, even before ISC to the ^3^MLCT and ^3^MC states has taken place. On a few-hundred fs time scale, the water molecules detached from the *axial* cyanides migrate to a position above/below the bipyridyl aromatic system, forming hydrogen bonds to the ligand that has increased electron density (*i.e.*, a negative charge) in the MLCT state. This constitutes a directed hydrogen bond migration mechanism without substantial involvement of bulk water. In contrast, water molecules recede from the *equatorial* cyanides into the second solvation shell to accommodate the changes in Fe–CN bond lengths.

We find that the solvent response is strongly damped, suppressing possible coherent oscillations in the solvent shells, even though coherent intramolecular vibrations occur in [Fe(CN)_4_(bipy)]^2−^. We also identified state-specific solvent distributions, which strongly differ between MLCT and MC states. While the MLCT response resembles the overall picture given in Fig. 2 and 3, the MC response only affects water molecules around the cyanide ligands, but not at the bipyridyl ligand. The characteristic solvent responses and the governing population in different electronic states are mutually influencing each other—the longer an electronic state stays populated, the more does the solvent reorganize in response to the state, which in turn stabilizes that state (and increases its lifetime) relative to other electronic states. In this case, the ^3^MLCT state's negative charge at the bipyridyl ligand is quickly stabilized by attracting nearby water molecules from the axial cyanide ligand present in the ground-state solvation shell. This highlights the critical influence of the solvent on the energetics and the nonadiabatic dynamics of [Fe(CN)_4_(bipy)]^2−^ in water. Applying the presented methodology to other solvents as well as other transition metal complexes will provide valuable insights into the subtle interplay between electronic states and solvent, deepening our understanding of solvation dynamics in photoactive transition metal systems.

## Data availability

The data supporting this article have been included as part of the ESI.†

## Author contributions

Severin Polonius: methodology, software, investigation, data curation, validation, visualization, formal analysis, drafting, writing – original draft, and writing – review and editing. Leticia González: conceptualization, funding acquisition, project administration, resources, discussion, writing – review and editing. Sebastian Mai: conceptualization, supervision, validation, visualization, formal analysis, writing – review and editing.

## Conflicts of interest

There are no conflicts to declare.

## Supplementary Material

SC-OLF-D5SC01174D-s001

SC-OLF-D5SC01174D-s002
