# Supplementary material for: Ultrafast solvent migration in an iron complex revealed by nonadiabatic dynamics simulations
Source: Chem Sci. 2025 May 13;16(24):11128–37. doi: 10.1039/d5sc01174d (PMC12100520; doi:10.1039/d5sc01174d)
Supplement: SC-016-D5SC01174D-s001 [file SC-016-D5SC01174D-s001.pdf]

Supplementary Information (SI) for Chemical Science.
This journal is © The Royal Society of Chemistry 2025

Supporting Information: Ultrafast Solvent Migration in an Iron Complex Revealed by Nonadiabatic Dynamics Simulations

Severin Polonius,[†,‡] Leticia González,[*,†] and Sebastian Mai[*,†]

†*Institute of Theoretical Chemistry, Faculty of Chemistry, University of Vienna, Währinger Str. 17, 1090 Vienna, Austria.*

‡*University of Vienna, Vienna Doctoral School in Chemistry (DoSChem), Währinger Str. 42, 1090 Vienna, Austria.*

E-mail: leticia.gonzalez@univie.ac.at; sebastian.mai@univie.ac.at

Contents

S1 Supplementary Computational Details **S-3**
S1.1 Electronic Structure Level of Theory and Geometry Optization S-3
S1.2 Parametrization of the VC Model S-5
S1.3 Generation of Initial Conditions S-7
S1.4 Excited State Dynamics S-7

S2 Supplementary Results and Discussion **S-10**
S2.1 Comparison with previous work S-10
S2.2 Selected Frames from the TD-3D-SDF Movie S-13
S2.3 Radial distribution functions S-14
S2.4 Ground and Excited State Atomic Charges S-18
S2.5 Hydrogen Bond Analysis S-19
S2.6 Time-Dependent 3D-SDF Slices S-20
S2.7 Migration Analysis of Water Molecules S-22
S2.8 Cartesian-weighted RDFs and X-ray scattering S-24

References **S-35**

S1 Supplementary Computational Details

The simulations presented in the main manuscript were generated in several steps. First, the level of theory was verified and a geometry optimization of $[FeCN_4bpy]^{2+}$ including frequency calculation was performed (Section S1.1). Second, a vibronic coupling model for $[FeCN_4bpy]^{2+}$ was generated based on the level of theory and the optimized reference geometry (Section S1.2). Third, using the AMBER package, classical molecular dynamics trajectories were ran to produce a set of 30,000 initial conditions for the system containing $[FeCN_4bpy]^{2+}$ and 5412 water molecules (Section S1.3). Subsequently, the initial conditions were stochastically excited to the bright singlet states and 4473 trajectories were propagated for 5000 fs using the VC/MM approach and the SHARC package (Section S1.4). Details and relevant references are given in the following subsections.

S1.1 Electronic Structure Level of Theory and Geometry Optization

For the sake of consistency, we used a comparable level of theory as in Ref. S1 to generate the parameters of the vibronic coupling (VC) model for $[FeCN_4bpy]^{2+}$. This includes a geometry optimization, frequency calculation, and all single-point calculations using the B3LYP* functional,[S2] and the def2-TZVP basis set for the iron center and the def2-SVP basis set for all other atoms. All calculations employed high convergence criteria with the SCF convergence set to $1 \cdot 10^{-10}$ au and the "superfine" grid option in GAUSSIAN.[S3] We additionally used the empirical dispersion correction GD3BJ defined in ORCA5.[S4,S5]

The solvation treatment in the computations deserves special mention: On one hand, the lowest excited states of$[FeCN_4bpy]^{2+}$ in the gas phase are close in energy to the ground state (excitation energies below 0.5 eV), which leads to convergence problems in the single-point calculations of the parametrization step later (see Fig. S1 for $\varepsilon_r = 1$). On the other hand, parametrizing the VC/MM model with an implicit solvent model (CPCM) with a dielectric constant of 80.2 (as appropriate for water) leads to double-counting of the solvent interactions once the explicit water molecules are introduced. Fig. S2 shows the resulting absorption spectrum for the case where solvent interactions are fully double-counted, leading to very bad agreement with the TDDFT reference.[S1] Hence, all electronic structure calculations were performed with CPCM considering only the high-frequency limit of the dielectric constant of water. This produces sufficiently high excitation energies for the first excited states (Fig. S1b), while avoiding the double-counting of solvent interactions.

We set the geometry optimization to be constraint to a C_{2v} symmetry for the molecular structure. At the obtained nuclear coordinates, we calculated the frequencies of $[FeCN_4bpy]^{2+}$ with the settings described above.

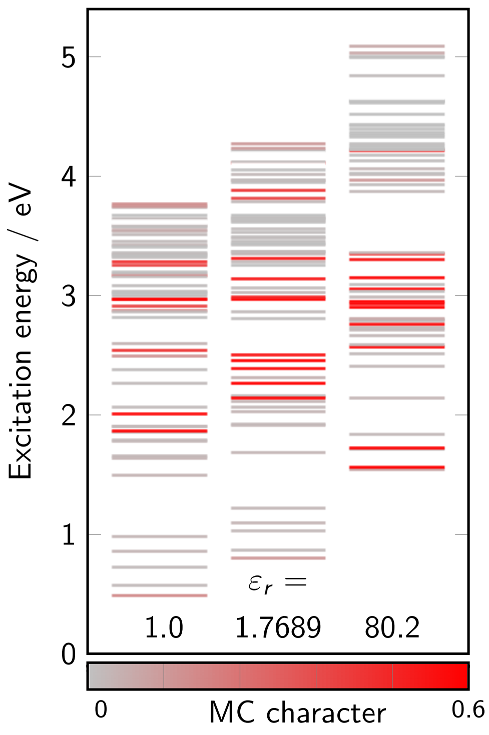

Figure S1: Excitation energies of $[FeCN_4bpy]^{2+}$ including implicit solvation with different dielectric constants ε_r. The shade indicates the metal-centered character of the respective excited state.

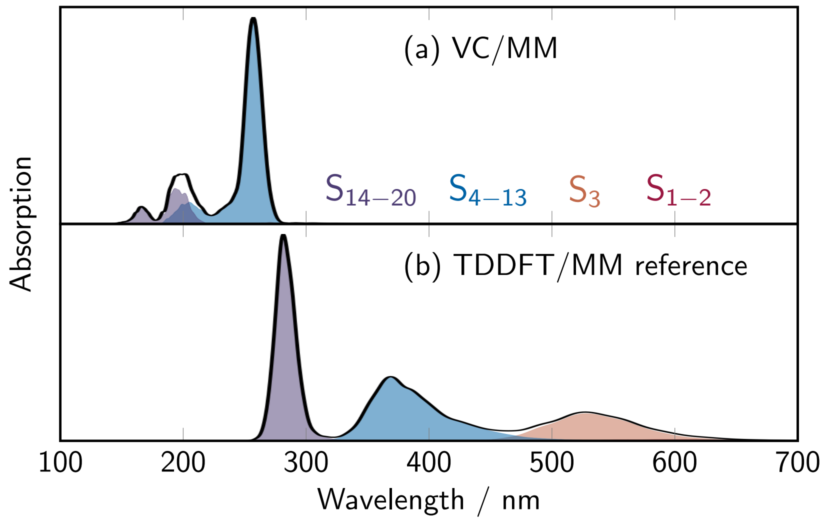

Figure S2: Spectrum of explicitly solvated $[FeCN_4bpy]^{2+}$ based on 500 initial conditions. Panel (a) shows the results of a VC/MM model parametrized with implicit solvation ($\varepsilon_r = 80.2$). Panel (b) shows the corresponding TDDFT/MM reference spectrum on the same initial conditions. The different absorption bands are highlighted by collecting the singlet states as they appear in the reference spectrum: S_1 and S_2 in red, S_3 in orange, S_4 to S_{13} in blue and S_{14} to S_{20} in purple. The initial conditions are provided in Ref. S1.

S1.2 Parametrization of the VC Model

With the computed geometry and normal modes of $[FeCN_4bpy]^{2+}$, we set up the calculation for the VC model according to Ref. S6. We decided to include the first 21 diabatic singlet and 20 diabatic triplet states in the model. This choice originated from overlap calculations including 31 singlets and 30 triplets with CPCM using an epsilon of 80.2 to gauge the behavior of the states in the solvated system. Analysis of the overlap and character of the states with the TheoDORE program[S7] revealed that the important six lowest MC triplet states can be represented within the first 20 triplet states with our CPCM settings.

For the numerical evaluation of the λ parameters, we performed single-point calculations at displaced geometries along each mass–frequency-weighted normal model scaled by 0.05 and 0.1. In this study, we extended the set of parameters of standard VC models (which typically include only linear terms)[S8] so that we can account for some state-specific shifts in frequency of selected normal modes including quadratic coupling terms. These parameters will be briefly explained here. In a general vibronic coupling model,[S6,S9,S10] the elements of the diabatic vibronic coupling matrix W_{ij} are constructed as a Taylor series expansion around the mass–frequency weighted normal coordinates $\mathbf{Q} = 0$

$$W_{ij} = \delta_{ij}\left(V_0(\mathbf{Q}) + \epsilon_i + \sum_k \kappa_k^{(i)} Q_k + \sum_{kl} \gamma_{kl}^{(ii)} Q_k Q_l + \cdots\right) + (1-\delta_{ij})\left(\eta_{ij} + \sum_k \lambda_k^{(ij)} Q_k + \sum_{kl} \gamma_{kl}^{(ij)} Q_k Q_l \cdots\right), \quad (1)$$

where

$$\kappa_k^{(i)} = \left(\frac{\partial W_{ii}}{\partial Q_k}\right)_{\mathbf{Q}=0} \quad (2)$$

$$\lambda_k^{(ij)} = \left(\frac{\partial W_{ij}}{\partial Q_k}\right)_{\mathbf{Q}=0} \quad (3)$$

$$\gamma_{kl}^{(ij)} = \left(\frac{\partial^2 W_{ij}}{\partial Q_k \, \partial Q_l} - \omega_k \delta_{kl}\right)_{\mathbf{Q}=0}. \quad (4)$$

Here, $V_0(\mathbf{Q}) = \frac{1}{2}\sum_k \omega_k Q_k^2$ is the ground state potential energy function with the frequency ω_k of the k-th normal mode, ϵ_i is the vertical excitation energy of the electronic state i at $\mathbf{Q} = 0$, η^{ij} are constant couplings parameters, $\kappa_n^{(i)}$ and $\lambda_n^{(ij)}$ are the first-order intrastate and interstate vibronic coupling constants, and $\gamma_{kl}^{(ij)}$ are the second-order coupling constants. Specifically, $\gamma_{kk}^{(ii)}$ are the state-specific frequency shifts that modify the curvature of the ground-state oscillator of mode k for state i, which we include in the VC model of $[FeCN_4bpy]^{2+}$ to account for the weakening of the Fe–X bonds in the MC states.

For the numerical evaluation of the γ parameters, we calculated additional single points at displacements 0.2 and 0.4 along all symmetric modes (13 modes) that elongate the Fe-N_{bpy} and Fe-$C_{eq.}$ bonds. This choice is motivated by Fig. S3, which shows that the inclusion of these parameters specifically increases the accuracy of the ^{3}MC states in the VC model. The

γ values are then calculated from these further displacements via as the mean as

$$\gamma_k^{(ij)} = \frac{2}{N}\sum_n^N \omega_{k,n} = \frac{2}{N}\sum_n^N \frac{\left[E^{(ij)}(h_{k,n}) - E^{(ij)}(0)\right]}{h_{k,n}^2}. \tag{5}$$

Here, $\omega_{k,n}$ is the evaluated frequency of normal mode k at displacement n and $E^{(ij)}(h_{k,n})$ is the diabatized energy at $h_{k,n}$ in normal mode coordinates. We only included $\gamma_{kk}^{(ii)}$ terms in the VC parameters for all states with major MC character at the equilibrium geometry and normal modes that affect the bond lengths between the metal center and equatorial ligands.

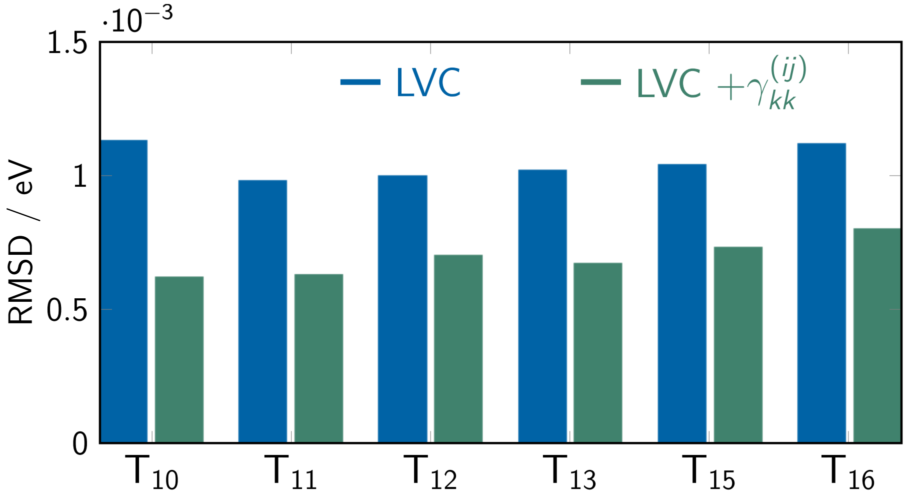

Figure S3: Root-mean-square deviations (RMSDs) of the excitation energies of triplet states with major metal-centered character computed with VC models (only linear terms denoted as LVC) against the TDDFT reference. The RMSDs are compared for the VC model with state-specific frequency shifts ($\gamma_{kk}^{(ii)}$) in green and without them in blue. The RMSDs are calculated from scans ($Q_k \in [-0.5, 0.5]$) of selected normal modes (11, 15, 19, 20, 31, 32, 47, and 71) that affect bond length between the iron center and equatorial ligands of $[FeCN_4bpy]^{2+}$.

To use a VC model in VC/MM simulations, we also prepared parameters for a distributed multipole expansion (DME) of all relevant electron densities. The DMEs are evaluated using the procedure outlined in Ref. S11 and restraint parameters of 0.001, 0.003 and 0.006 for the multipole orders with target charges for the monopolar terms set to 0 and van-der-Waals radii as recommended in Ref. S12. For the simulations, we fitted DMEs for each diabatic the electronic states and included them in the VC model.

The spin–orbit coupling parameters included in the VC model are calculated using ORCA5,[S5] with settings equivalent to the GAUSSIAN calculations. The RMSD of the adiabatic energies between GAUSSIAN and ORCA was 0.0010 au (30 meV) and a electronic states showed consistent wave function character and oscillator strengths between the two programs.

S1.3 Generation of Initial Conditions

The initial conditions for the excited state dynamics are generated using both MD and VC/MM calculations. The procedure is adapted from both Ref. S13 and Ref. S1. For the MD simulations using AMBER22,[S14] we use the parameter file containing a custom-built force field for $[FeCN_4bpy]^{2+}$ from Ref. S1. We modified this file by updating the charges of $[FeCN_4bpy]^{2+}$ with the fitted monopole charges from the DME of the ground state. As a water model, we used SPC-Fw.[S15]

To build the system, the iron complex was solvated in a truncated-octahedron water box containing 5412 water molecules and two Na^+ ions with 25 Å between the complex and the closest face of the box. After relaxing the generated solvation box to remove bad contacts/voids, we used molecular dynamics simulations with a time step of 0.5 fs to heat the system to 300 K over 20 ps (NVT ensemble) and equilibrate it to 1 bar over 500 ps (NPT ensemble). Finally, a production run of 45 ns was performed, from which we took snapshots every 1.5 ps to obtain 30,000 initial conditions.

In order to approximately account for the zero-point energy of the complex that cannot be described with nuclear dynamics, we increased the kinetic energy of $[FeCN_4bpy]^{2+}$ through a local reheating step, as proposed in Refs. S13 For the local reheating step, the atomic positions of the solvent nuclei were kept fixed using the 'ibelly' option of `AMBER`,[S14] and the solute complex was reheated to 600 K over 40 ps. Afterward, the velocities of the solvent molecules were reset to the values before freezing. This generates a system in which the solute molecule has kinetic energy approximately equivalent to 600 K, while the solvent is at 300 K.

At this point, we deviated from the original procedure[S1] to some degree, as the efficiency of the VC/MM method allows for a longer re-equilibration. All 30,000 initial conditions with the solvent at 300 K and the solute at approximately 600 K were re-imaged to place the iron complex at the center of the solvent box. The system was then re-equilibrated in the ground state using SHARC[S16] and VC/MM, propagating for a random time between 500 and 550 fs. During the re-equilibration run, we saved the nuclear coordinates of the last 100 fs every 5 fs, to provide data for the ground state solvent distribution that is employed as reference in the solvent resonse analyses later.

S1.4 Excited State Dynamics

The initial conditions were stochastically excited[S17] at the first absorption band in a window between 482 nm to 502 nm (2.52 ± 0.1 eV) according to the calculated spectrum (Fig S4a) shown below. This resulted in the excitation of 4473 initial conditions into the lowest 5 singlet excited states with a majority (4366, 97.6%) in the S_3 state.

The trajectory swarm was set up using all 4473 excited initial conditions. The trajectories were propagated with the surface hopping including arbitrary couplings (SHARC) method,[S18] using a local development version of the SHARC package.[S11,S16,S19] In the simulations, we used the local diabatization approach to propagate the electronic wave function,[S20,S21] the energy-based decoherence correction,[S22] rescaling of the solute atom velocities after a hop parallel to their velocity vector, and no special treatment of frustrated hops. In the SHARC simulation, the lowest six adiabatic singlet and seven adiabatic triplet states were used for the dynamics; these states were computed from the full VC model including 21 diabatic

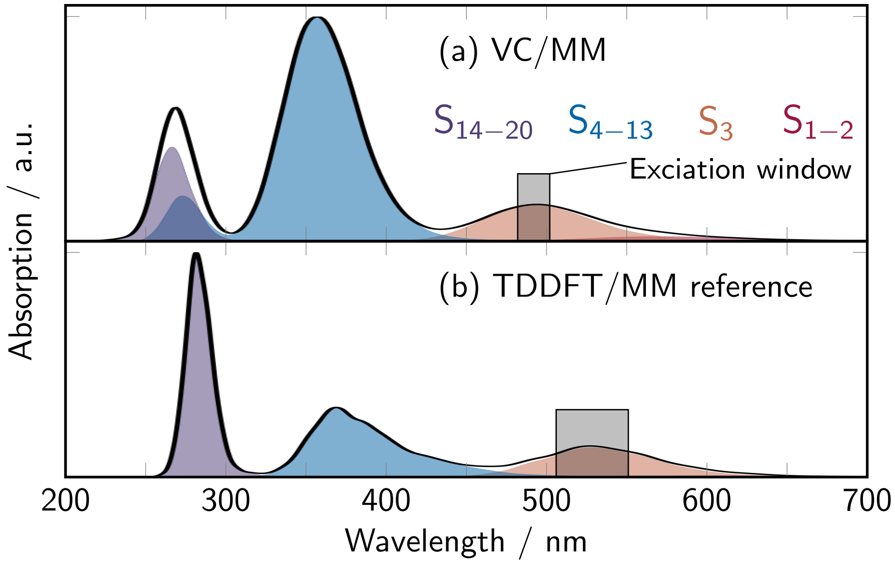

Figure S4: Absorption spectra of $[FeCN_4bpy]^{2+}$ in water computed with (a) the VC/MM model from 30,000 initial conditions and (b) the TDDFT/MM reference method from 500 initial conditions provided by the authors of Ref S1. The different absorption bands are highlighted by collecting the singlet states as they appear in the reference spectrum: S_1 and S_2 in red, S_3 in orange, S_4 to S_{13} in blue and S_{14} to S_{20} in purple. Both spectra are convoluted with Gaussian distribution functions (full-width-half-maximum of 0.1 eV) and normalized to 1. The gray shaded area indicates the used excitation window.

singlet and 20 diabatic triplet states. The propagation time step was set to 0.02 fs for the electronic wave function and to 0.5 fs for the nuclear positions and velocities. All trajectories were propagated for 5000 fs. Each trajectory was computed on a single Intel Xeon E5-2650 v3 CPU and had an average wall time of 13 h; this corresponds to about 4.7 seconds per step (or 2.6 CPUh/ps). For comparison, the TDDFT/MM simulations from Ref. S1 consumed about 4 CPUh per time step (8,000 CPUh/ps); thus, in the previous work only 99 trajectories were simulated for only 700 fs.

Data on the electronic wave function (Hamiltonian, overlap and transformation matrices) were stored every 1 fs for times up to 700 fs and then every 5 fs (1560 data points); the nuclear coordinates were saved every 5 fs for times up to 700 fs and then every 25 fs (312 data points). The complete dataset on electronic and nuclear data for the whole trajectory swarm amounted to about 1.1 TiB. The electronic dynamics were analyzed with respect to the electronic populations and the characters of the excited states. The characterization of the electronic states was performed in the spin-free adiabatic basis and in the diabatic basis. The latter can be accessed without loss of information because the VC model is defined in the diabatic basis and the full transformation matrices of all steps are known. The diabatic populations are then interpreted based on the charge transfer characters of the reference states (computed at the reference geometry using the TheoDORE program[S7]). The total singlet and triplet populations as well as the diabatic populations were fitted using tools from the SHARC program[S16] with either a monoexponential ansatz or a specified kinetic model (as shown below and in Fig. 1 in the main text). Errors for the obtained time-constants were

computed via bootstrapping.[S23]

S2 Supplementary Results and Discussion

S2.1 Comparison with previous work

This work is closely related to that published in Ref. S1, which reported on-the-fly SHARC TDDFT/MM excited-state dynamics of $[FeCN_4bpy]^{2+}$ in water. Thus, a brief comparison between both works is in order. The results from Ref S1 will be referred to as "TDDFT reference" henceforth.

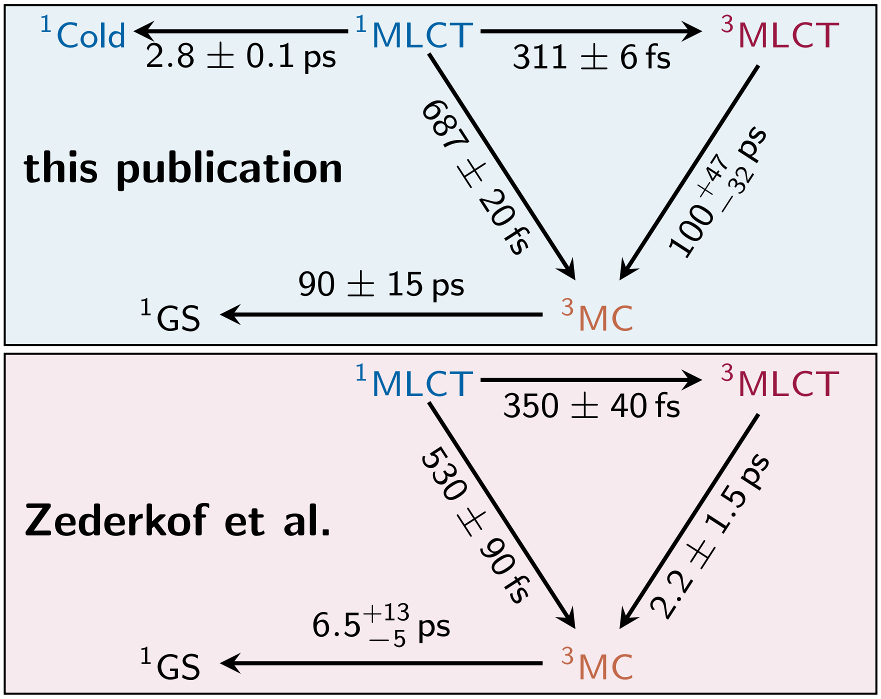

Figure S5: Comparison of kinetic models of the solvated excited state dynamics of $[FeCN_4bpy]^{2+}$ between this work (top) and the work of Zederkof et al.[S1] (bottom). The kinetic model of this work is in the diabatic basis with the manifolds of different multiplicity and electronic character summed up. The kinetic model of the Zederkof et al.[S1] is in the adiabatic basis with contributions to manifolds of different multiplicities and electronic characters summed up.

Comparing the electronic results in Fig. 1 with the TDDFT reference (Fig. S5), evidences an excellent agreement in terms of ISC rate (210 ± 4 fs vs 211 ± 20 fs). Also, the individual time scales for the conversion from ^{1}MLCT to ^{3}MLCT (310 fs with VC/MM, 350 fs with TDDFT/MM) and to ^{3}MC (690 fs with VC/MM, 530 fs with TDDFT/MM) are reasonably reproduced. However, the time scales for the slower conversion processes are significantly longer in VC/MM than in TDDFT/MM. Specifically, the conversion from ^{3}MLCT to ^{3}MC has a 100^{+47}_{-32} ps time constant in VC/MM and a 2.2 ± 1.5 ps time constant in TDDFT/MM. For the relaxation from ^{3}MC to the ground state, VC/MM gives 90 ± 15 ps, whereas TDDFT/MM gives 6.9^{+13}_{-5} ps. We note that these differences do not arise from the differences in the used kinetic models (in the present work, the kinetic model contains the extra ^{1}Cold species) or the differences in simulation time (5 ps in VC/MM versus 0.7 ps in TDDFT/MM).

The time constant for the relaxation into the ground state reported in the TDDFT reference (6.9^{+13}_{-5} ps) is consistent with the experimentally determined ones of about 13 ps.[S24] Hence, the VC/MM model appears to be limited in the accuracy of the description of the ^{3}MLCT–^{3}MC and ^{3}MC–ground state conversions, which are known to be controlled by the stabilization of the involved ^{3}MC states.[S24] It is thus likely that the ^{3}MC states are not stabilized enough in the VC/MM model compared to the other triplet states, by either limitations in their solvent interactions or their intramolecular potential energy surface.

The time-dependent excitation energies of the diabatic states are shown in Fig. S6, separated into MC (orange) and MLCT (red) character. As can be seen, the MC states are consistently slightly below the MLCT states. Nonetheless, the MC and MLCT energies are so similar that no large driving force for fast MLCT→MC dynamics is present.

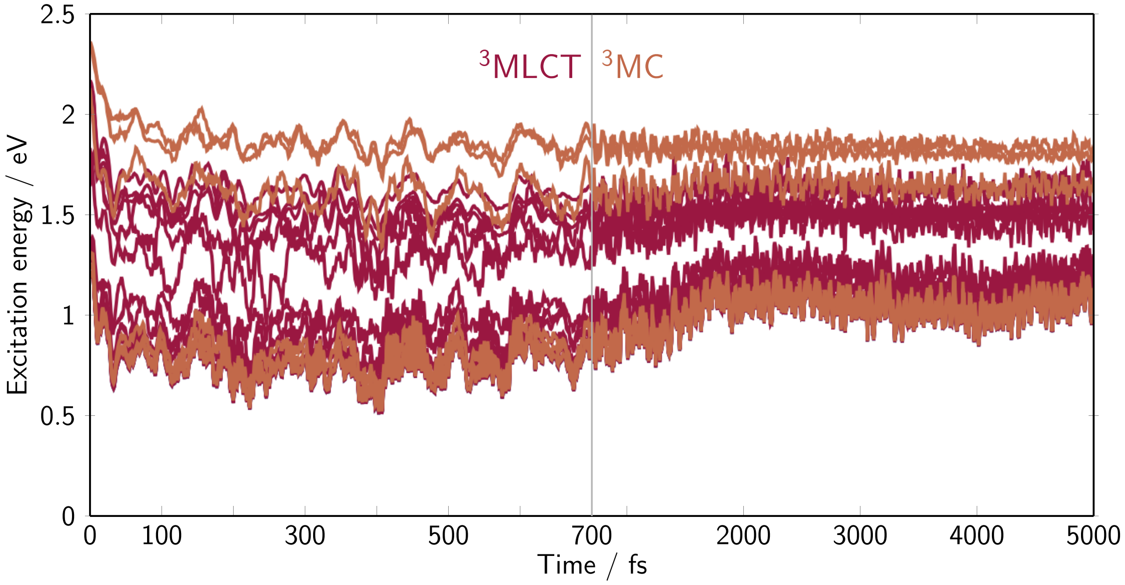

Figure S6: Average diabatic excitation energies of metal-centered (MC) and metal-to-ligand-charge-transfer (MLCT) triplet states in orange and red over time.

Fig. S7 shows the time-dependent distributions of bond length of the Fe–X bonds, weighted by MC (red) and MLCT (blue) character, with the ground state at $t < 0$ (black). Comparing these bond length distributions with the TDDFT reference (Fig. 3 in Ref. S1), we find that, even though the average bond lengths are well reproduced, the shape and width of the distributions differ. The TDDFT reference distributions of the Fe–N_{bpy} and Fe–CN_{eq} in the ^{3}MC states are broad and appear to be skewed towards longer bond lengths. This indicates significant anharmonicities in the normal modes describing the Fe–X distances. The respective distributions in the VC model are both symmetric and less broad (see Table S1), as the VC model—despite the inclusion of some state-specific quadratic terms—cannot represent the anharmonicity of these bonds. We, thus, conclude (i) that the inclusion of additional second-order terms is definitely necessary and has shown merit for a better description of the system and (ii) that terms which account for anharmonicity are relevant for this system, so that the PESs of the MC states can be accurately modeled.

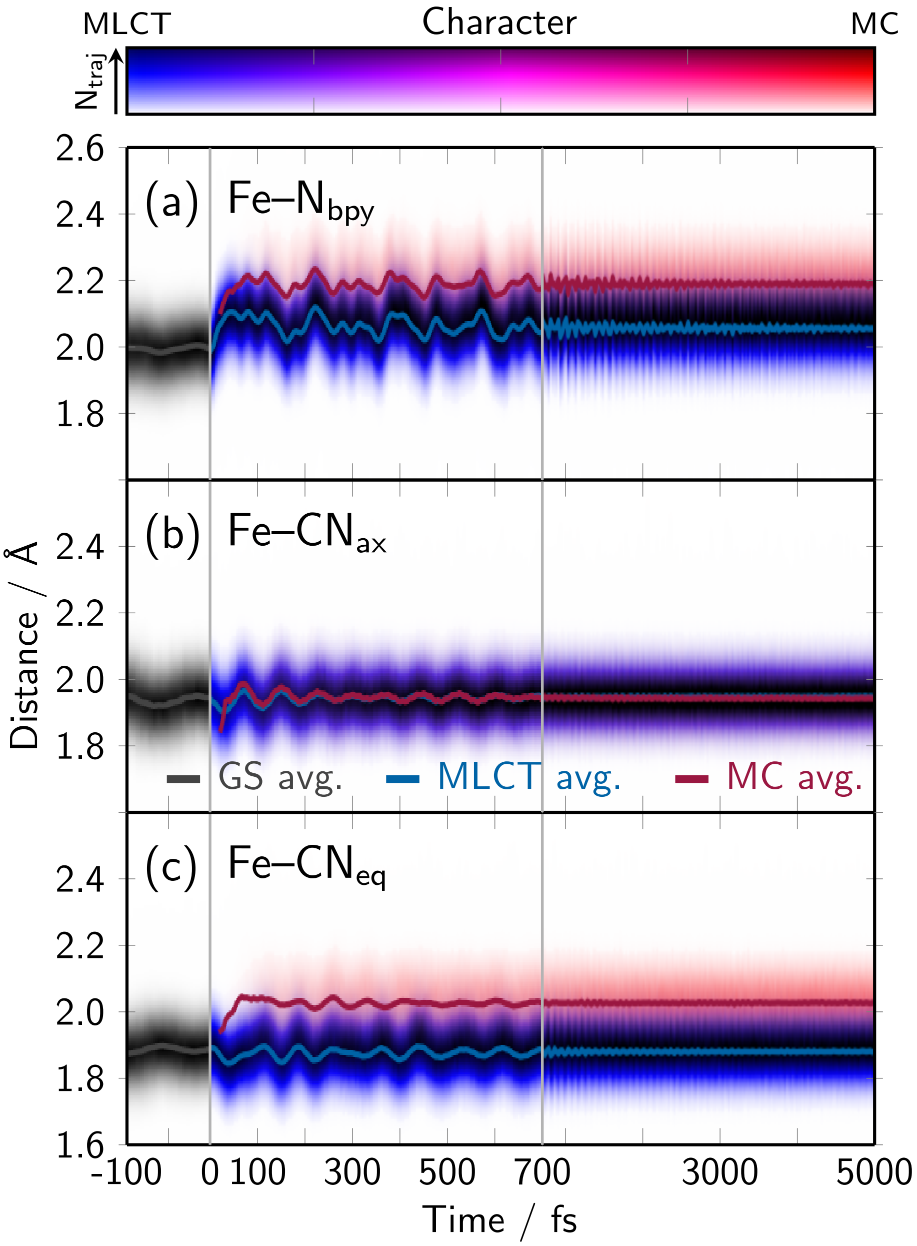

Figure S7: Distributions of different bond lengths over time decomposed into the contribution of electronic states with different character. Both the averages (lines) and distributions (shaded areas) are shown for the iron to bipyridyl nitrogen bond length (Fe–N$_{bpy}$) in panel a, the iron to axial cyanide carbon bond length (Fe–CN$_{ax}$) in panel b, and the iron to equatorial cyanide carbon bond length (Fe–CN$_{eq}$) in panel c. The ground state is indicated by black and gray colors. The contributions to the bond lengths of the excited states with different character are indicated by red for MC states to purple for a mixture to blue for MLCT states. The luminance of each color decreases with the number of corresponding trajectories with active MC or MLCT state.

Table S1: Root-mean-square values of positive deviations ($RMSD_+$) and negative deviations ($RMSD_-$) from the mean bond length value after excitation shown in Fig. 3.

	MLCT		MC	
	$RMSD_+$	$RMSD_-$	$RMSD_+$	$RMSD_-$
Fe–N_{bipy}	0.069	0.070	0.080	0.082
Fe–CN_{ax}	0.066	0.064	0.065	0.063
Fe–CN_{eq}	0.063	0.062	0.070	0.073

S2.2 Selected Frames from the TD-3D-SDF Movie

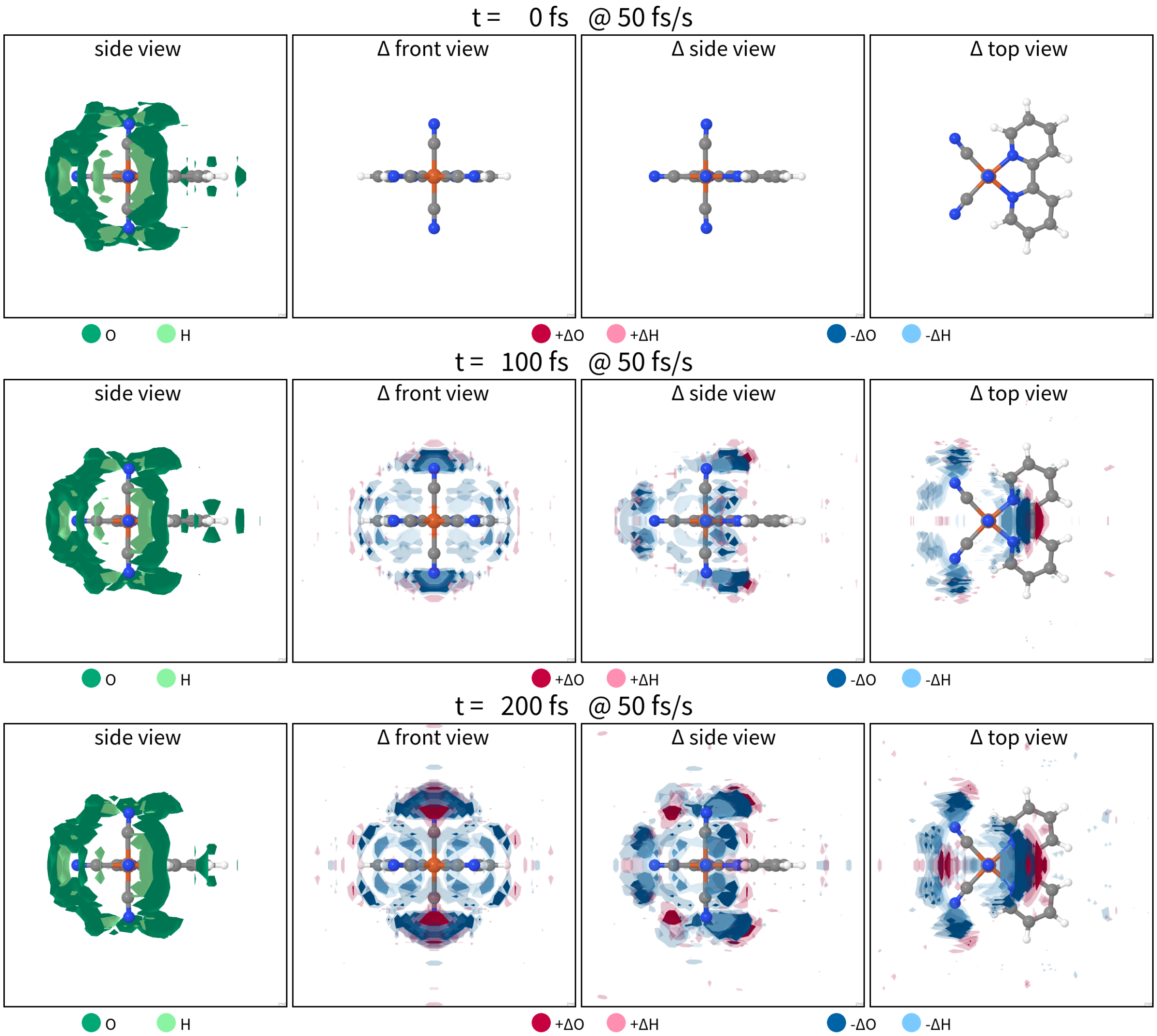

Figure S8: Frames from movie of symmetry-adapted 3D-SDFs and Δ3D-SDFs equivalent to Fig. 2 in the main manuscript.

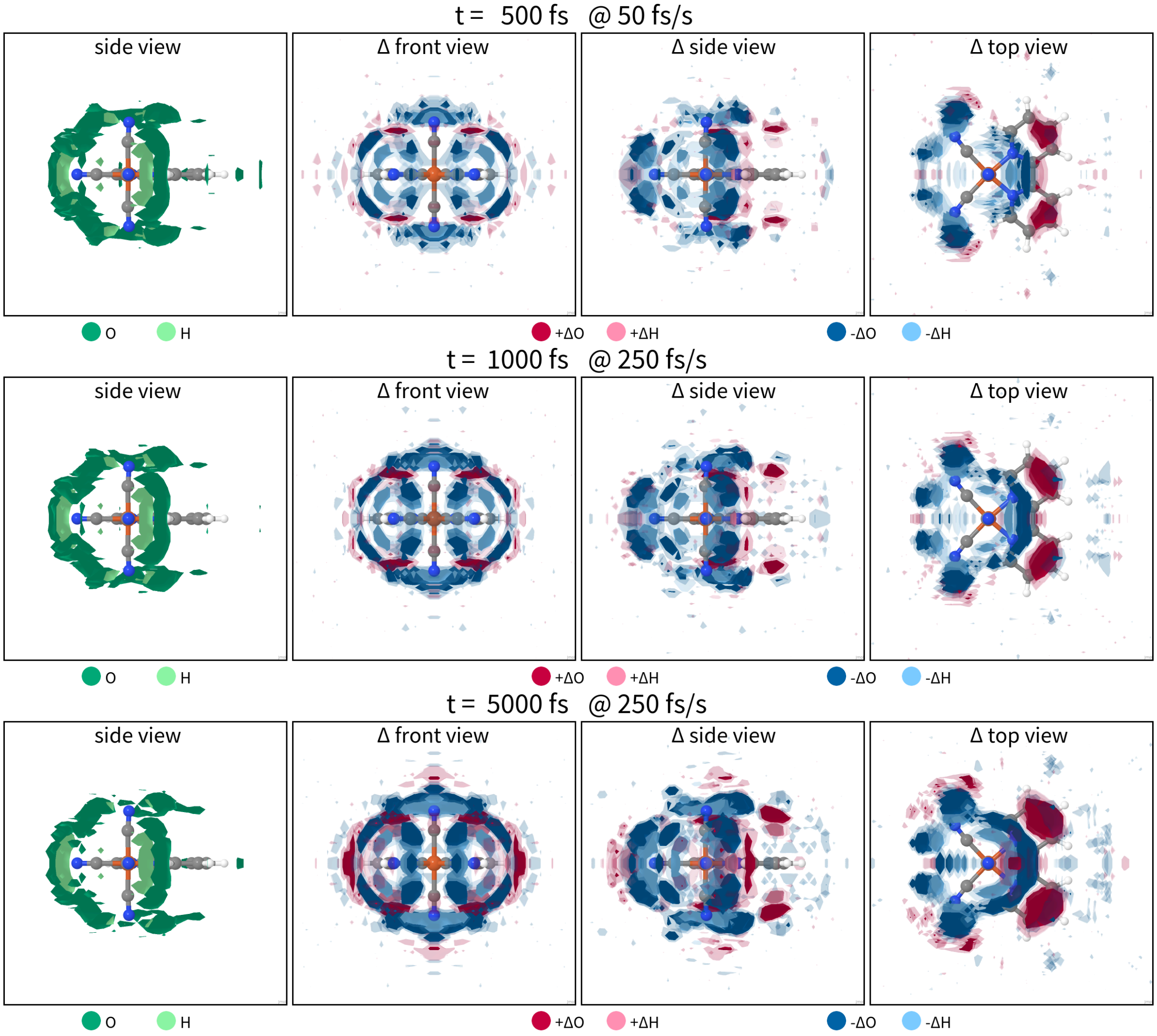

Figure S9: Frames from movie of symmetry-adapted 3D-SDFs and Δ3D-SDFs equivalent to Fig. 2 in the main manuscript.

S2.3 Radial distribution functions

Radial distribution functions (RDFs) were calculated between different pairs of atom collections and are displayed in Fig. S10. All RDFs are evaluated with bin widths of 0.05 Å and normalized by $\frac{4\pi}{3}\left[(R+\mathrm{d}R)^3-R^3\right]$ using the `cpptraj` program of the AmberTools program package.[S14] The average RDFs from −100 to 0 fs before excitation serve as a reference to compute difference RDFs (ΔRDFs) over time. The temporal behavior of the ΔRDFs is then analyzed by calculating singular value decompositions (SVDs) as $\Delta\mathrm{RDF}(R,t)=\sum_i V_i(R)\cdot s_i\cdot U_i(t)$ and fitting the temporal components $U_i(t)$.

Fig. S10 shows the RDFs of C_{bpy}–H_{sol}, C_{CN}–H_{sol}, and N–H_{sol} in panels (a-c) respectively. It can be seen that C_{bpy}–H_{sol} does have a comparably weak first solvation shell. However, the RDF at $t=700$ fs shows an significant increase in water hydrogen atoms at distances between 2 and 3 Å. The other two RDFs show strong first solvation shells at about 2.5Å for C_{CN}–H_{sol}

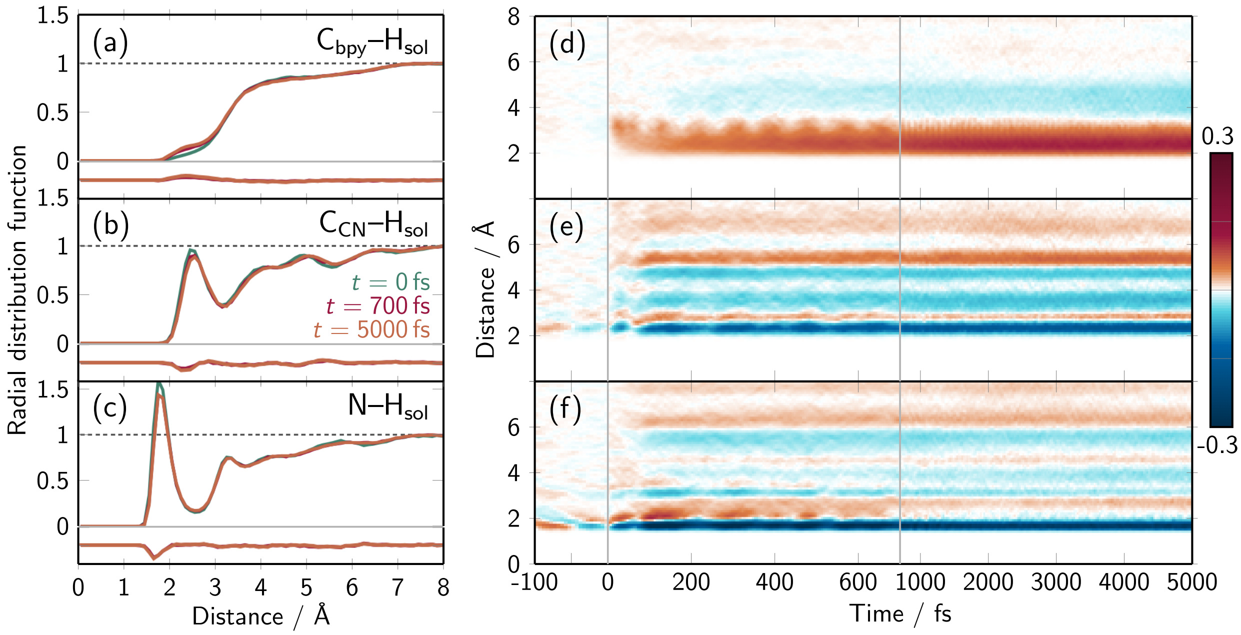

Figure S10: Radial distribution functions (RDF) at different times after excitation (a-c) and corresponding time-dependent difference RDFs (d-f). (a-c) Show the bipyridyl carbon–water hydrogen (C_{bpy}–H_{sol}), cyanide carbon–water hydrogen (C_{CN}–H_{sol}) and nitrogen–water hydrogen (N–H_{sol}) RDFs at $t = 0$ fs in green, $t = 700$ fs in red and $t = 5000$ fs in orange (the respective differences are shown below 0). Panels (d-f) show the respective time-dependent difference RDFS corresponding to (a-c) with positive deviations in red colors and negative deviations in blue colors.

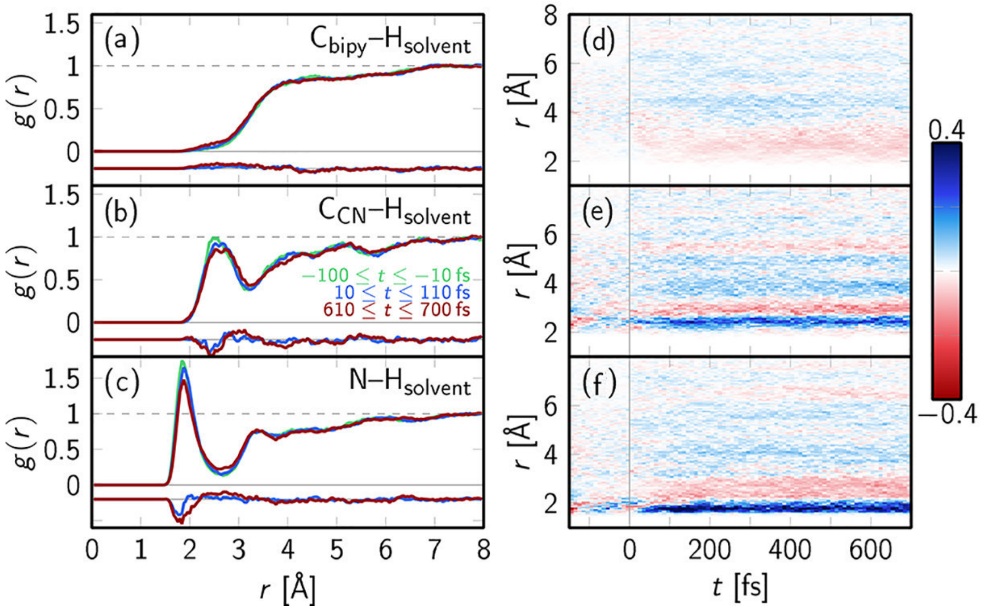

Figure S11: RDFs (a–c) and time-dependent difference RDFs (d–f) based on 99 TDDFT/MM trajectories analogous to Fig. S10. Reproduced from Ref. S1. Copyright ©2022 The Authors.

and about 1.9 Å for N–H_{sol}. Both signals decrease within the first 700 fs after excitation and remain at approximately the same level until the end of the simulation. We note that these RDFs agree excellently with the ones shown in Ref.[S1] in Fig. 4 (partially reproduced in Fig. S11), including all visible extrema and shoulders and their temporal behaviour.

Fig. S10d shows an interesting oscillating feature in the C_{bpy}–H_{sol} RDF at around 2–4 Å. The signal initially rises with a 277 fs time constant (monoexponential fit of $U_1(t)$) and stabilizes at later times. The oscillation period is about 88 fs, which corresponds to a frequency of 378 cm^-1. This oscillation can be assigned to a symmetric stretch mode of the bipyridyl ligand, which stretches/compresses the entire ligand along its long axis. In particular, the average of the distances between symmetry-equivalent bipyridyl carbon and nitrogen atoms shows a period of 87 fs. The remarkable influence of this oscillation on the RDF agrees with previous findings,[S1] although here we can resolve the oscillations with much lower noise level and for longer time scales.

The time-dependent C_{CN}–H_{sol}, and N–H_{sol} RDFs in Fig. S10e–f can be used to quantify the time scale of the initial solvent response after excitation. Using monoexponential fits of $U_1(t)$, we obtain time scales of 73 fs and 47 fs from panels e and f, respectively (from Ref. S1: 56 ± 14 fs and 41 ± 8 fs). Here, it is interesting to observe that the response of the RDFs propagates outwards rather quickly, as even at distances of 6–8 AA, the excitation response can be observed within less than 100 fs. Furthermore, we note that also these two time-dependent RDFs show (somewhat more difficult to discern) oscillations. For C_{CN}–H_{sol} (panel e), these are present around 2.5 Å with a period of about 150 fs, whereas for N–H_{sol} (panel f), they are located around 1.8 Å with a similar period.

In Fig. S12, we separate the RDFs of the equatorial and axial cyanide ligands to observe differences in their dynamics. Based on panels a and b, it can be seen that the axial cyanides show a stronger response in their hydrogen bonds (2–3 Å), but rather small changes in the second solvent shell. In contrast, the equatorial cyanides show a weaker hydrogen bond decrease, but a stronger difference signal between 4 and 6 Å, indicating different dynamics in the second solvation shell. In panels c and d, it can be seen that the axial cyanide response starts immediately at $t = 0$, whereas the equatorial cyanides' solvent shell only starts responding around 60–100 fs. In the same panels, weak oscillations in the hydrogen bonding can be seen—for the equatorial cyanides, around 2.4 Å one can observe oscillations with a 160 fs period. For the axial cyanides, at the same distance, oscillations with 80 fs period can be observed instead. We note that an analysis of the equatorial and axial cyanide solvent shell dynamics was already attempted in Ref.,[S1] but due to the small amount of trajectories, no conclusive distinctions could be found.

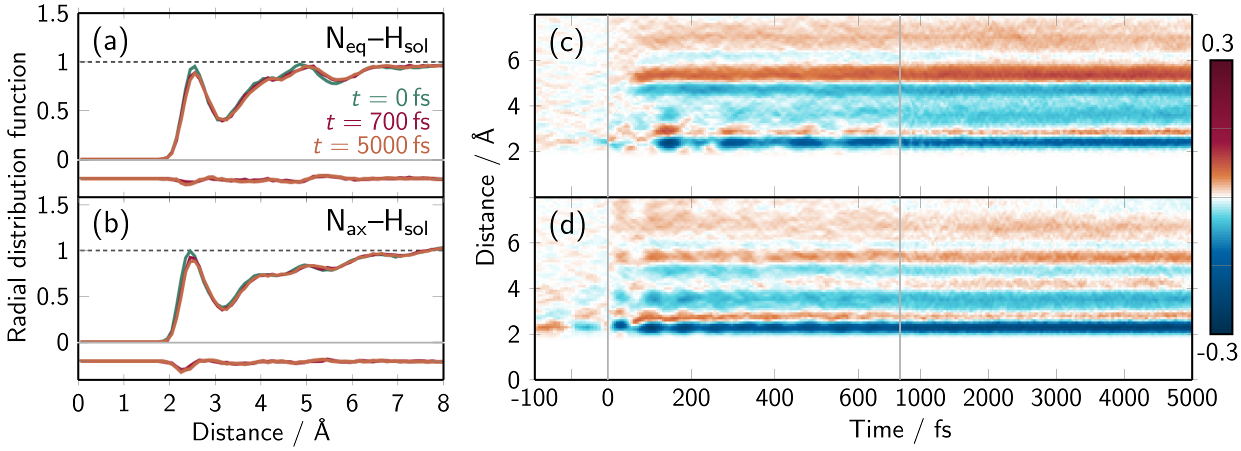

Figure S12: Radial distribution functions (RDF) at different times after excitation (a-b) and corresponding time-dependent difference RDFs (c-d). (a-b) Show the axial and equatorial cyanide nitrogen–water hydrogen RDFs (N_{ax}–H_{sol} and N_{eq}–H_{sol}) at $t = 0$ fs in green, $t = 700$ fs in red and $t = 5000$ fs in orange (the respective differences are shown below 0). Panels (c-d) show the respective time-dependent difference RDFS corresponding to (a-b) with positive deviations in red colors and negative deviations in blue colors.

S2.4 Ground and Excited State Atomic Charges

The changes in the atomic charges for the excited states with respect to the ground already allow a distinction between the MLCT and MC states: States that with only minor changes in the atomic charges have major metal-centered character (S_{10},S_{13},S_{14}, and T_{10},T_{11},T_{12},T_{13},T_{15},T_{16}) and the other states have major metal-to-ligand-charge character with most states increasing the charge, and hence electronic density, on the bipy ligand.

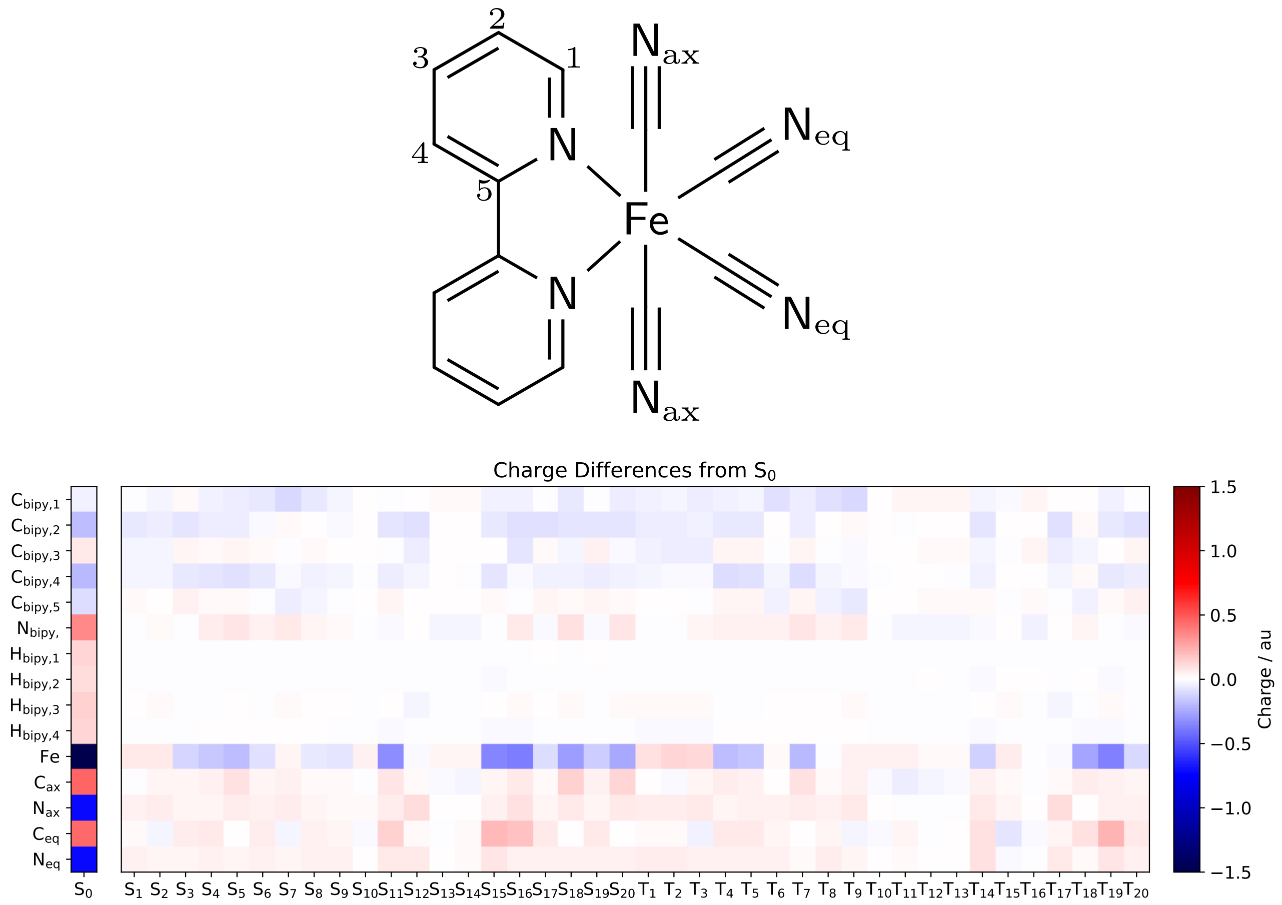

Figure S13: RESP[S11,S25]-fitted atomic charges for the ground state (left most column) and difference from the ground state for all excited states (center columns) for all atoms at the ground-state equilibrium geometry fitted. The labeling is shown at Lewis structure on top and symmetrically equal atoms are grouped.

S2.5 Hydrogen Bond Analysis

Fig. S14 shows time-dependent hydrogen bond counts for N_{CN} and C_{bpy}, based on a range of distance criteria and a 135° angle criterion. The overall hydrogen bond counts at both sites are drastically different, with N_{CN} having an about ten times higher count than C_{bpy}. Independent of the distance criterion, the figure reproduces the finding that the initial solvent response leads to a decrease in N_{CN}–H–O counts and an increase C_{bpy}–H–O counts; the latter increase is continued further at a slower pace. Bi-exponential fits of the curves with a cutoff of 4.2 Å provide time constants of $\tau_1(N_{CN}) = 14$ fs and $\tau_1(C_{bpy}) = 240$ fs for the initial response and $\tau_2(N_{CN}) = 32$ fs and $\tau_2(C_{bpy}) = 495$ fs for the secondary response; this cutoff value was chosen because the curves show a drastic change at $t = 0$ fs showing the libration motion of the water molecules. The newly formed hydrogen bonds at the C_{bpy} atoms represent a stark increase with respect to the ground state and stabilize the long-lived MLCT states.

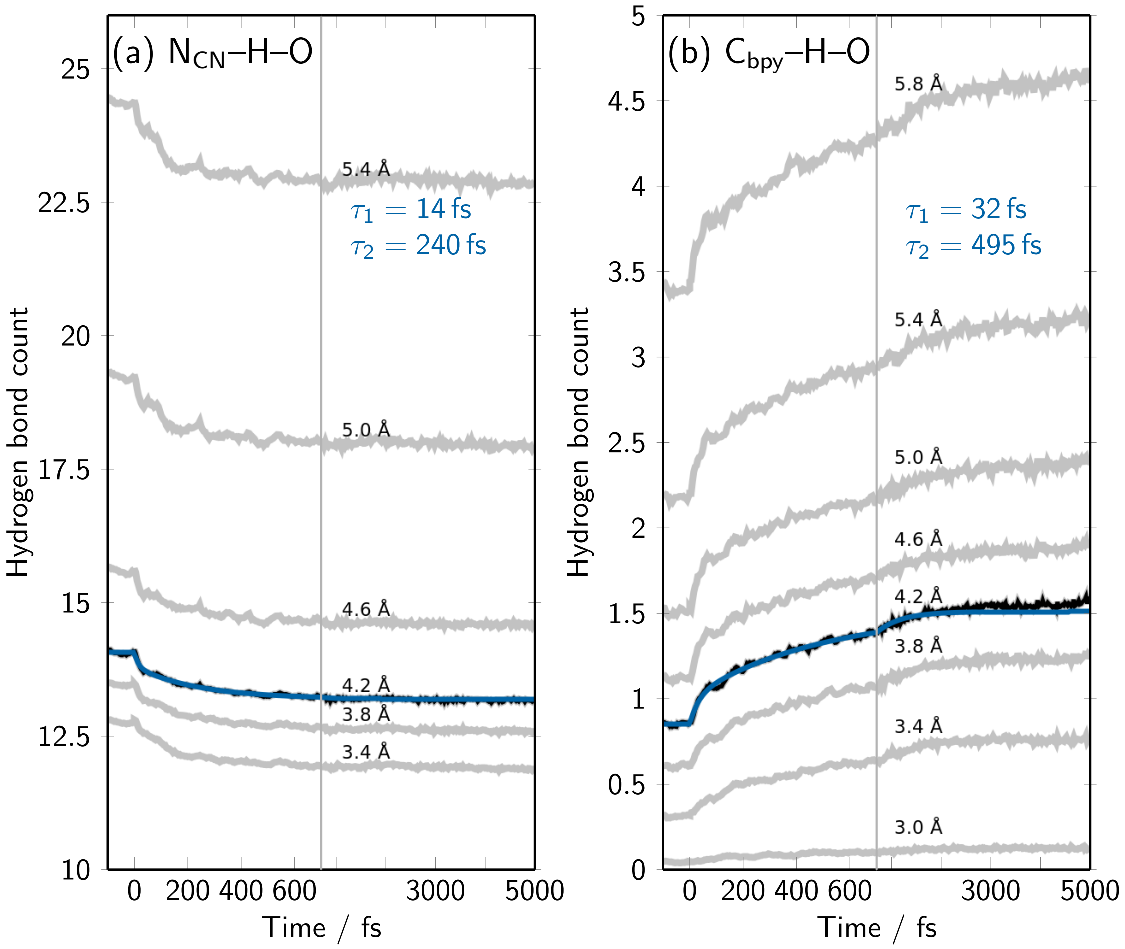

Figure S14: Time-dependent hydrogen bond counts averaged over all 4473 trajectories determined from geometric criteria scanned over the distance cutoff; the angular cutoff is kept at 135°. Panel (a) shows the hydrogen bond counts for the cyanide nitrogen atoms and panel (b) the counts for the bipyridyl carbon atoms. A bi-exponential fit (blue line) for a cutoff of 4.2 Å (black curve) results in the two time constants given in the plot.

S2.6 Time-Dependent 3D-SDF Slices

Figs. S15 and S16 show slices through the time-dependent difference 3D-SDF (for oxygen atoms) in three Cartesian directions. For each of the two figures, the three slices intersects at a common point, as indicated in the panels at the top. These figures help visualizing density fluctuations in more detail than in the three-dimensional isovalue plots. The figures show that the solvent shell does not exhibit any coherent oscillations, but the solvent evolution is smooth and relatively strongly damped. This is relevant, because Fig. S10 shows significant coherent oscillations in several solute–solvent RDFs. The slices in Figs. S15 and S16 show

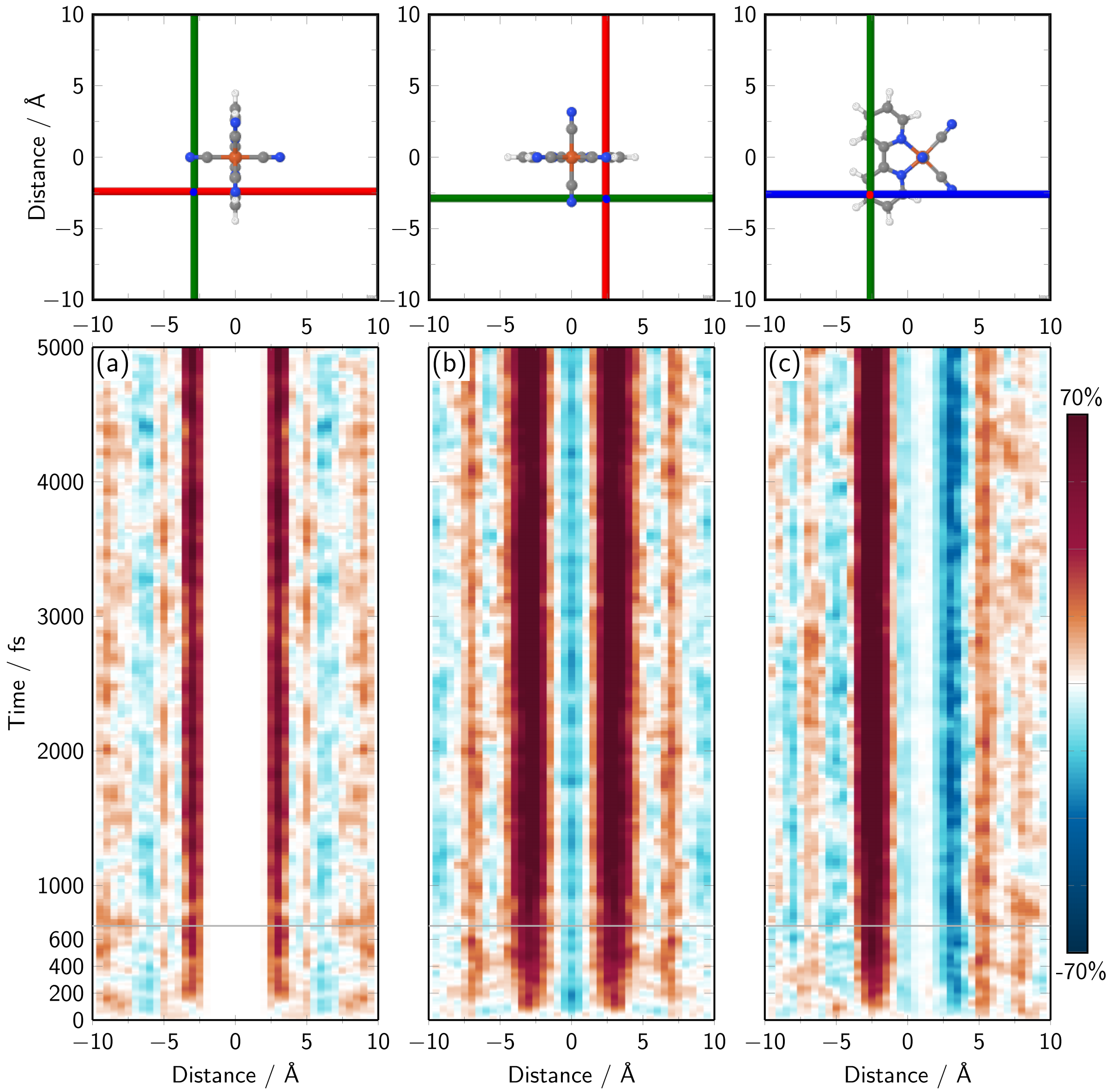

Figure S15: Time-dependent 3D-SDF slices (oxygen atoms). Each panel (a-c) shows the time dependence of one row of pixels from the 3D-SDF: panel a shows the pixels along the red line, panel b for the green line, panel c for the blue line.

clearly that these oscillations do not originate from coherent fluctuations in the solute.

In more detail, Fig. S15 is centered on the location where the hydrogen bonds to the bipyridyl ligand accumulate. The slice in panel a is effectively in terms of the distance from the bipyridyl plane. Oxygen accumulation starts around 200 fs and tapers off around 1000 fs. Panels b and c show the same oxygen accumulation (strong red colors) with other surroundings.

The slices in Fig. S16 are centered on the place above the C–C bridge where the oxygen briefly accumulates after acceleration. This can be seen in the red feature around 100–300 fs in all three panels. Again, no oscillations are visible.

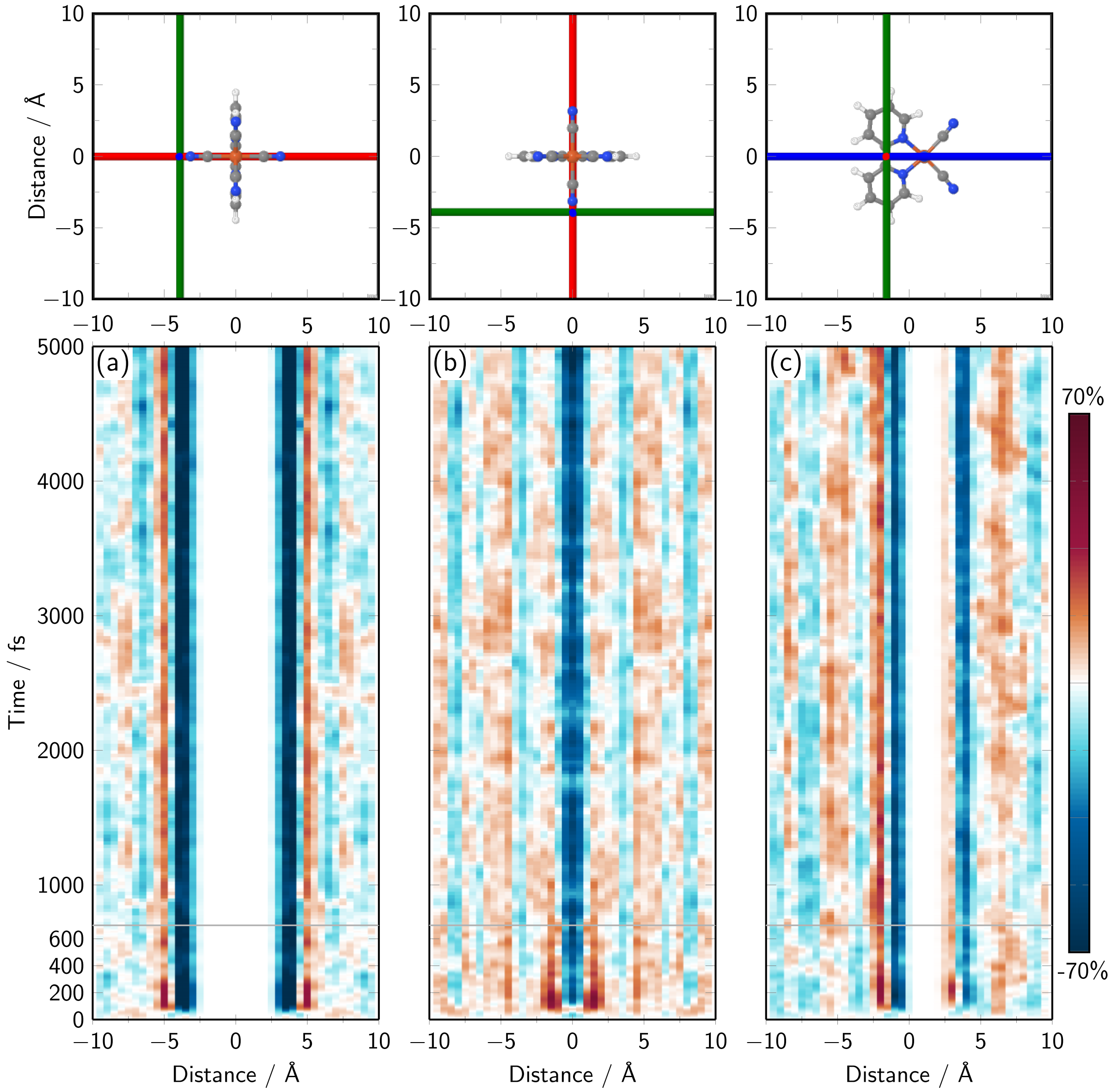

Figure S16: Same as Fig. S15, but for a different intersection point.

S2.7 Migration Analysis of Water Molecules

For the water migration analysis, the 3D space around the molecule was discretized into 0.5 Å grid cells (like for the 3D-SDFs) and then eight different regions were defined, as shown in Fig. S17. The regions were chosen based on the raw data of the 3D-SDFs at $t = 0$ fs and $t = 5000$ fs, to define regions that represent the first solvent shells around the axial and equatorial cyanides and the bipyridyl, the second solvation shells around cyanides and bipyridyl, and the bulk. The "far bulk" region includes all space outside of the $40 \times 40 \times 40$ grid. For the migration analysis, we then counted the numberO_{ij} of all oxygen atoms that were in region i at $t = 0$ fs and ended in region j at $t = 5000$ fs.

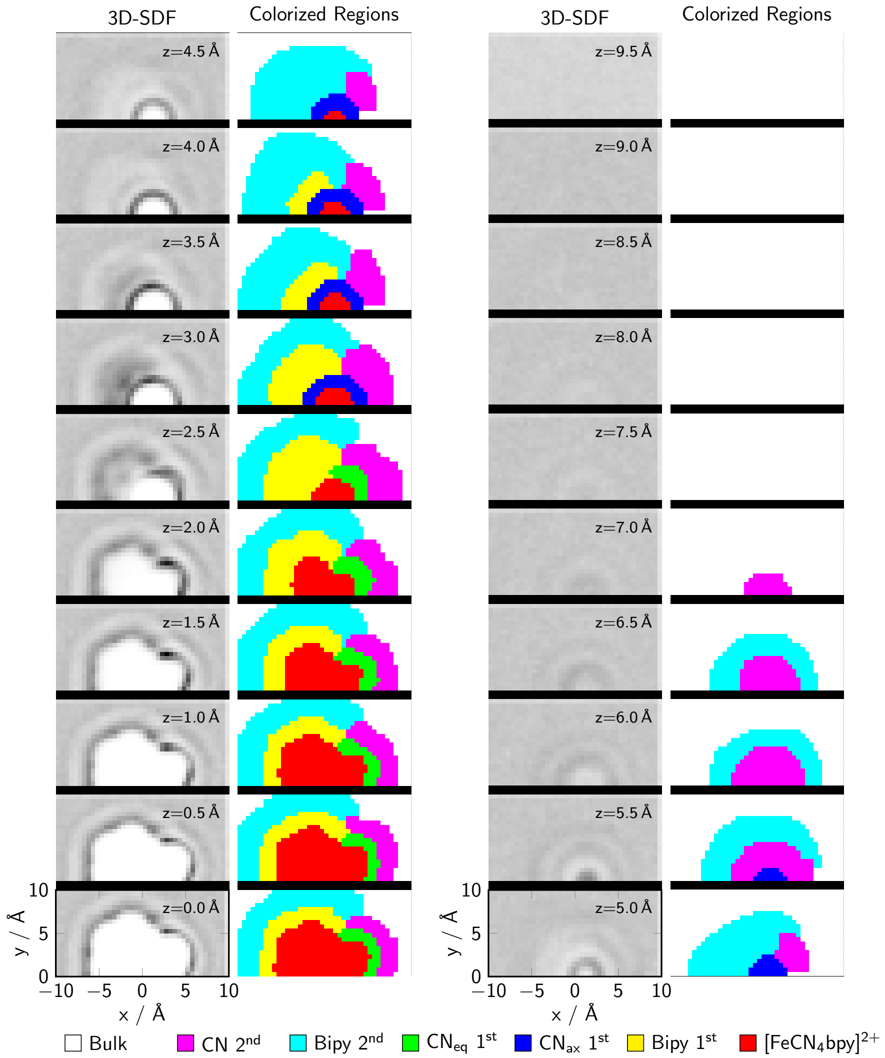

Figure S17: Definition of solvent shell regions around $[FeCN_4bpy]^{2+}$. The grey-scale columns show slices through the average of the $t = 0$ fs and 5000 fs 3D-SDFs. The colored columns show the definition of the regions: bulk water (white), CN second solvation shell (magenta), bipy second solvation shell (cyan), CN_{eq} first solvation shell (green), CN_{ax} first solvation shell (blue), bipy first solvation shell (yellow), and the cavity of $[FeCN_4bpy]^{2+}$ (red).

The results are shown in Table S2. The analysis showed that, overall, the number of water molecules in each region does not change dramatically, due to the incompressibility of water and despite the fact that significant changes in the solvent shells were observed in the 3D-SDFs. The largest changes are +0.6 water molecules in the "bipy 2nd" region and −0.5 waters in the CN_{eq} region. Generally, the first solvation shell loses nearly one water molecule, and the second solvation shell gains about the same amount. The most important directed migration paths were from CN_{ax} to bipy ($1.1 - 0.8 = 0.3$ waters move in this direction), from CN_{eq} to CN 2nd ($1.7 - 1.4 = 0.3$ waters), and from bipy to bipy 2nd ($7.4 - 7.0 = 0.4$ waters). Hence, waters lost from equatorial cyanides move to the cyanide second solvation shell, whereas waters lost from axial cyanides end up coordinating the bipyridyl. Some of these waters coordinating the bipyridyl displace other waters into the bipyridyl second solvation shell. No strong/directed exchange of waters between the first solvation shell and the bulk were found. Consequently, the correlation analysis shows that hydrogen bond reorganization around the complex is primarily happening though direct migration of waters over the surface of the molecule, rather than through a bulk exchange mechanism, where waters from broken hydrogen bonds move into the bulk, and new hydrogen bonds are formed with waters originating from the bulk.

Table S2: Correlation matrix of the number of water molecules that start in one region of the solvation shell (rows) and end in another region (columns)

		**Waters ending in**								
		far bulk	bulk	CN 2nd	bipy 2nd	CN_{eq}	CN_{ax}	bipy	mol	Initial
**Waters starting in**	far bulk	5088.4	57.0	0.7	6.7	0.0	0.0	0.3	0.0	5153.1
	bulk	56.6	73.2	6.3	13.8	0.3	0.3	0.9	0.0	151.4
	CN 2nd	0.7	6.2	8.1	2.7	**1.4**	1.1	0.7	0.0	20.9
	bipy 2nd	6.9	13.7	2.6	20.2	0.2	0.5	**7.0**	0.0	51.1
	CN_{eq}	0.0	0.3	**1.7**	0.3	3.3	0.7	0.4	0.0	6.7
	CN_{ax}	0.0	0.3	1.1	0.6	0.6	2.9	**1.1**	0.0	6.6
	bipy	0.3	0.9	0.7	**7.4**	0.4	**0.8**	11.7	0.0	22.2
	mol	0.0	0.0	0.0	0.0	0.0	0.0	0.0	0.0	0.0
	Final	5152.9	151.6	21.2	51.7	6.2	6.3	22.1	0.0	
	Initial	5153.1	151.4	20.9	51.1	6.7	6.6	22.2	0.0	
	Change	−0.2	+0.2	+0.3	+0.6	−0.5	−0.3	−0.1	0.0	

Example: 1.7 waters started in the "CN_{eq}" region and ended in the "CN 2nd" region. "Initial" gives the sum of rows, "Final" the sum of columns, and "Change" the difference between Initial and Final. The total number of waters is 5412. Bold numbers indicate the region pairs where exchange was the most asymmetric/directed.

S2.8 Cartesian-weighted RDFs and X-ray scattering

To investigate the possibility of obtaining different X-ray scattering signals in different directions, we computed distance histograms weighted by the Cartesian vector components.

$$h^x_{AB}(R,t) = \frac{1}{N_{\text{traj}}} \sum_T^{N_{\text{traj}}} \sum_{a\in A} \sum_{b\in B} \delta\left(\left|\vec{R}^T_a(t) - \vec{R}^T_b(t)\right| - R\right) \frac{(x^T_a(t) - x^T_b(t))^2}{\left|\vec{R}^T_a(t) - \vec{R}^T_b(t)\right|^2} \tag{6}$$

is the distance histogram weighted by the squared x component of each individual distance vector $\vec{R}^T_a(t) - \vec{R}^T_b(t)$. N_{traj} is the number of trajectories and T is the index running over the trajectories. $h^x_{AB}(R,t)$ can be interpreted as the component of the RDF that is weighted with $\cos^2(\alpha)$, where α is the angle between the x axis and the distance vector. Analogous equations were used for the y and z components. Hence, the sum of the three Cartesian-weighted histogram components recovers the total histogram, $h^x_{AB}(R,t) + h^y_{AB}(R,t) + h^z_{AB}(R,t) = h_{AB}(R,t)$. The time-dependent difference RDFs plotted below were computed by normalizing the histograms:

$$g_{AB}(R,t) = \frac{h_{AB}(R,t)}{N_A(N_B - \delta_{AB})\frac{4}{3}\pi(R^3 - (R+\mathrm{d}R)^3)V}. \tag{7}$$

The plotted difference RDFs in Figs. S18, S19, S20, and S21 were obtained by subtracting the RDF at $t = 0$ from the RDF at time t. The same approach was used for the Cartesian-weighted RDFs. We note that the Cartesian-weighted histograms and RDFs were computed from the dynamics snapshots that were aligned to a reference geometry, as described above for the generation of the 3D-SDFs.

The simulated X-ray scattering signals shown below were obtained from:[S1,S26]

$$\Delta S_{AB}(Q,t) = f_A(Q) \cdot f_A(Q) \cdot \int_0^{R_{\text{cutoff}}} \left(h_{AB}(R,t) - h^{\text{ref}}_{AB}(R,t)\right) \cdot \frac{\sin(QR)}{QR}\,\mathrm{d}R. \tag{8}$$

Analogously, we transformed the Cartesian-weighted components using the same equation. The integral was evaluated with R_{cutoff} of 10 Å and a Q range between 0.01 and 15 Å^{-1}. The requisite atomic structure factors $f_A(Q)$ are computed as linear combinations of Gaussians, as described in Ref. S27:

$$f_A(Q) = c_A + \sum_{i=1}^{4} a_A \exp\left(-b_A\left(\frac{Q}{4\pi}\right)^2\right). \tag{9}$$

The scattering signals computed from the Cartesian-weighted histograms can be viewed as approximations to the X-ray scattering observed with polarized UV pump and X-ray probe experiments, where the molecules are preferentially excited when their transition dipole moment $\vec{\mu}$ is aligned with the pump polarization vector $\vec{E}$. If the wave vector $\vec{k}$ of the X-ray beam is parallel to the pump polarization vector/transition dipole moment ($\vec{k} \parallel \vec{\mu}$),[S28,S29] one will then predominantly observe scattering due to the distribution functions orthogonal to the molecular transition dipole moment.[S30,S31] For $[FeCN_4bpy]^{2+}$, the transition dipole moment of the bright S_3 is along the symmetry axis, and therefore X-ray scattering in an

$\vec{k} \parallel \vec{\mu}$ arrangement should be preferentially sensitive to dynamics orthogonal to the symmetry axis. Additional information can be gained with an $\vec{k} \perp \vec{\mu}$ arrangement, with exciting into a bright state with different polarization direction, or by aligning functionalized $[FeCN_4bpy]^{2+}$, e.g., in surface films.[S32]

Figs. S18, S19, S20, and S21 show the time-dependent difference RDFs corresponding to all solute–solvent cross-term element combinations (solute Fe, N, C, and H with solvent H and O). Fig. S10f is equivalent to Fig. S19 bottom left, and the sum of Fig. S10d and e is equivalent to Fig. S20 bottom left. In addition to the total time-dependent difference RDFs, Figs. S18, S19, S20, and S21 also show the Cartesian-weighted components of these RDFs. The Cartesian components visualize the solvent dynamics occurring around the molecule. In the used convention, the z axis is the symmetry axis that passes through the Fe atom and the bipyridine C–C bridge. The bipyridine ligand lies in the yz plane, and the x axis is orthogonal to the bipyridine plane and parallel to the axial cyanide ligands.

In Fig. S18, the main differences in the Cartesian components can be seen at 3 Å for H atoms and at just below 4 Å for O atoms. Here, it can be seen that along the z axis, some O atoms approach the Fe atom, whereas this does not occur to the same extent in the x and y directions.

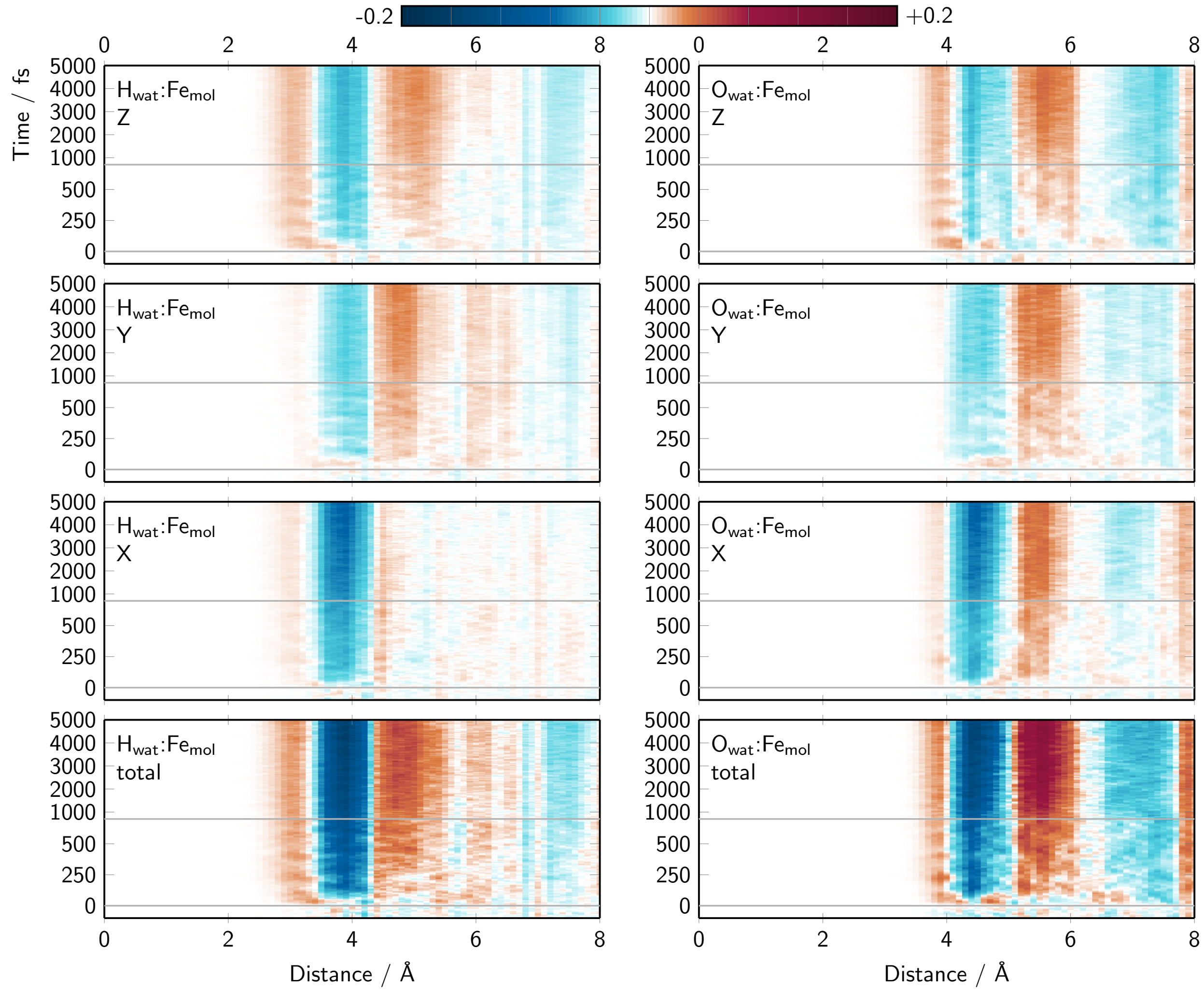

Figure S18: Time-resolved difference RDFs between Fe atoms of the molecule and H atoms (left) or O atoms (right) of the solvent. The bottom panels show the total difference RDFs, whereas the panels above show the Cartesian components of the RDFs in x, y, and z direction (see labels). Note that the x, y, and z components add to the total difference RDFs and that all panels are plotted with the same normalization.

In Fig. S19, for the RDFs related to the N atoms, the differences between the Cartesian components are also relatively small. This can be understood from the fact that the four cyanides are surrounded in all three Cartesian directions by hydrogen bonds, which exhibit similar dynamics. Upon closer inspection, one can see that the x component is weaker than the y and z components. We assume this is due to the hydrogen bonds of the axial cyanide ligands lying in the same yz plane as the cyanide N atoms, and therefore the solvent dynamics around the axial cyanides does not contribute to the x component of the difference RDFs.

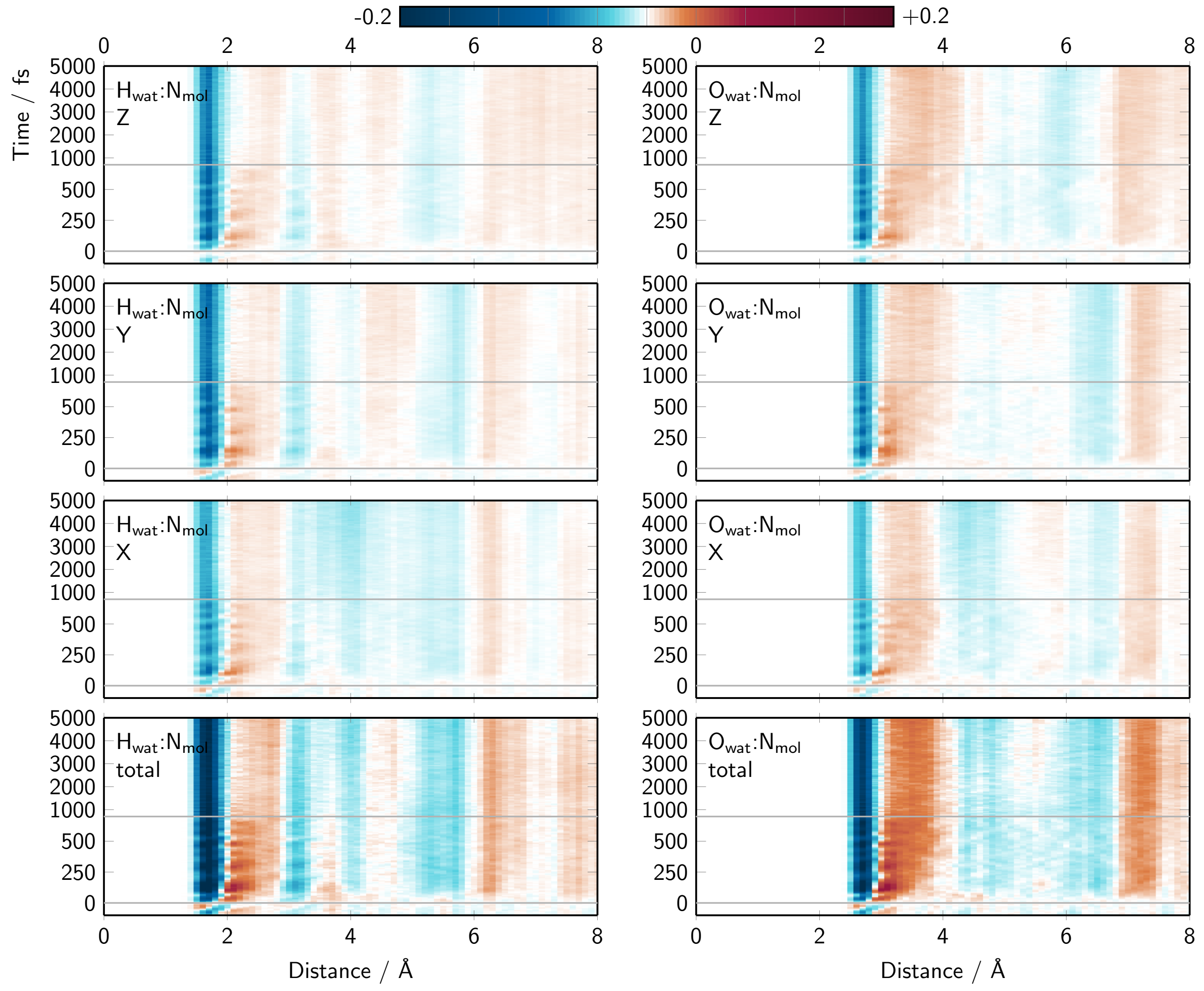

Figure S19: Time-resolved difference RDFs between N atoms of the molecule and H atoms (left) or O atoms (right) of the solvent. The bottom panels show the total difference RDFs, whereas the panels above show the Cartesian components of the RDFs in x, y, and z direction (see labels). Note that the x, y, and z components add to the total difference RDFs and that all panels are plotted with the same normalization.

In Fig. S20, the difference RDFs related to the C atoms are shown, exhibiting the largest dissimilarities across the Cartesian components of the difference RDFs. For the C–O RDFs, the x component shows a significant increase at about 3 Å and a decrease around 4 Å. This feature is due to the formation of hydrogen bonds to the C-rich bipyridine ligand in the MLCT state. In contrast, in the y and z components, a weak depletion at around 3 Å can be observed, stemming from the dynamics around the cyanide ligands. The y component also exhibits the coherent oscillations due to the bipyridine stretching mode discussed in the main text. The Cartesian components of the RDFs of the carbon atoms encode the most useful information to observe the anisotropic dynamics around the molecule.

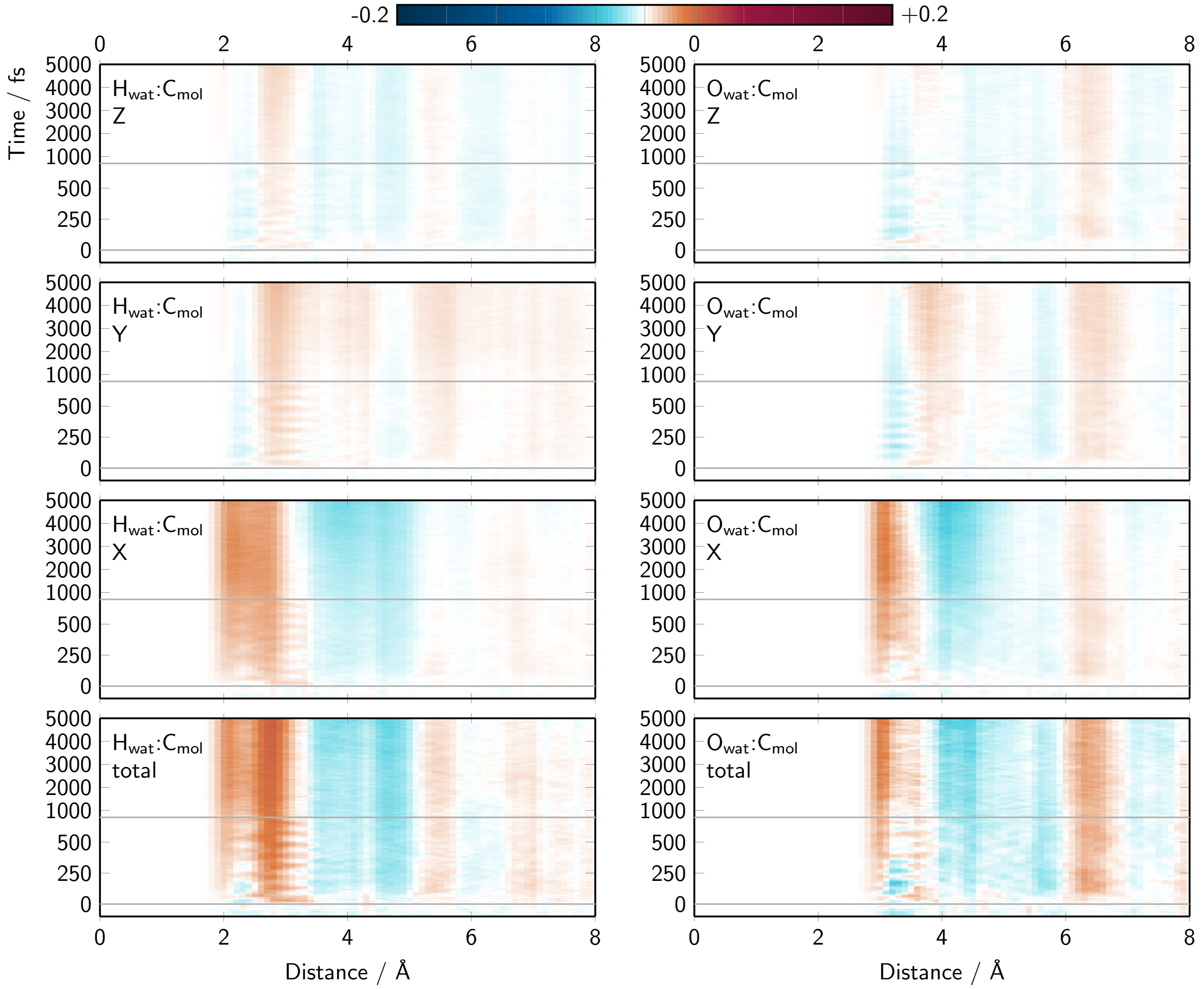

Figure S20: Time-resolved difference RDFs between C atoms of the molecule and H atoms (left) or O atoms (right) of the solvent. The bottom panels show the total difference RDFs, whereas the panels above show the Cartesian components of the RDFs in x, y, and z direction (see labels). Note that the x, y, and z components add to the total difference RDFs and that all panels are plotted with the same normalization.

In Fig. S21, the difference RDFs for the solute H atoms also show some differentiation between the x and y and z components. However, the observed features are due to the same processes as in the N and C RDFs discussed before. Additionally, the RDFs related to the H atoms are not very relevant for the X-ray scattering signals.

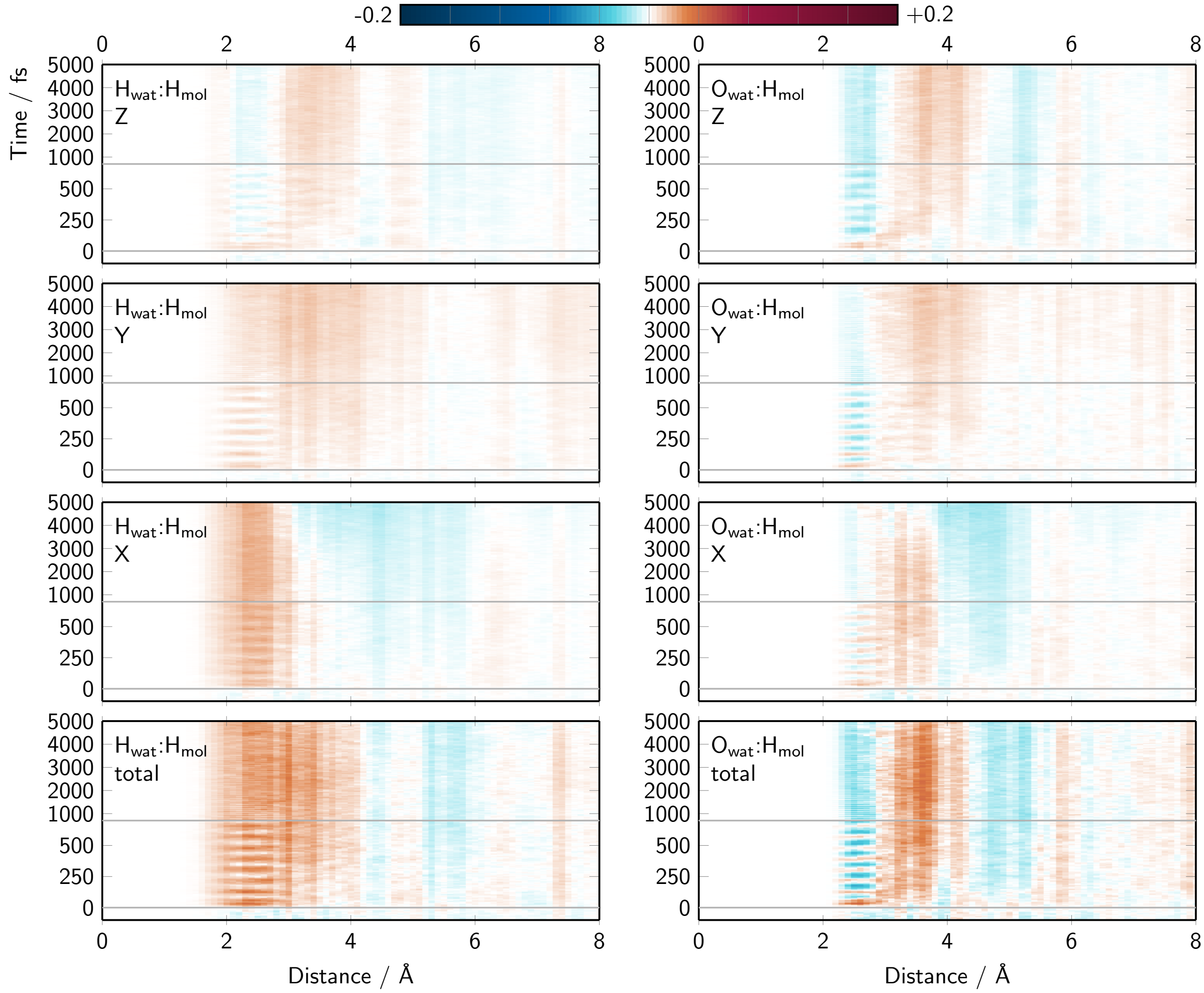

Figure S21: Time-resolved difference RDFs between H atoms of the molecule and H atoms (left) or O atoms (right) of the solvent. The bottom panels show the total difference RDFs, whereas the panels above show the Cartesian components of the RDFs in x, y, and z direction (see labels). Note that the x, y, and z components add to the total difference RDFs and that all panels are plotted with the same normalization.

Figs. S22, S23, S24, and S25 show the time-resolved difference scattering signals due to Fe, N, C, and H atoms of the solute. Fig. S26 finally shows the total scattering signals. These signals contribute to the solute–solvent cross term; we did not compute the solute term or the solvent term in this work. Note that the three contributions can be separated experimentally due to their different concentration and temperature dependencies.[S1]

Fig. S22 exhibits relatively strong signals from the Fe–O scattering, whereas—as expected—the Fe–H scattering is weak. As in the RDFs, some differences can be observed between the x component and the y and z components.

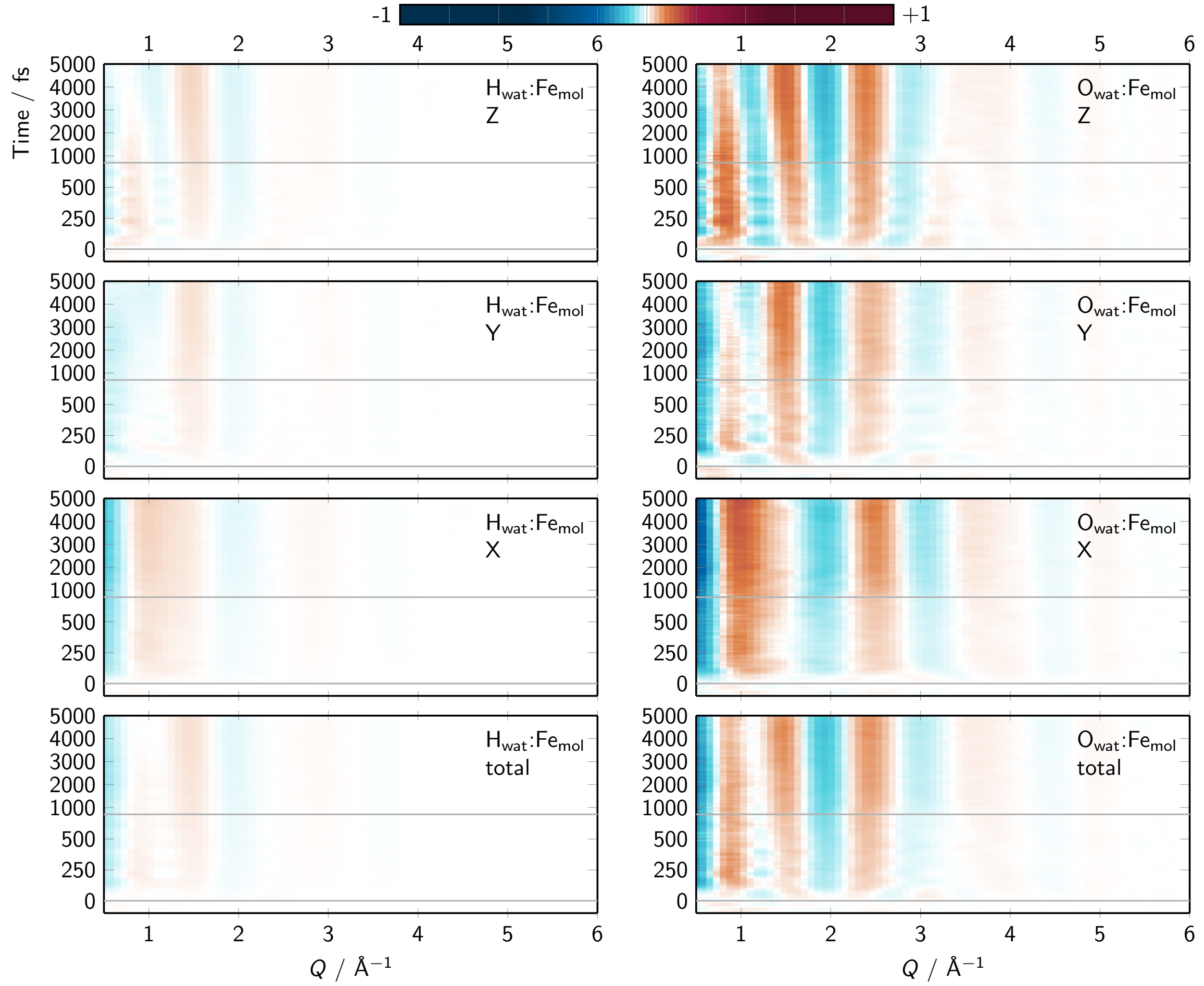

Figure S22: Time-resolved difference scattering due to Fe atoms of the molecule and H atoms (left) or O atoms (right) of the solvent. The bottom panels show the total difference scattering, whereas the panels above show the scattering due to the Cartesian components of the RDFs in x, y, and z direction (see labels). Note that the x, y, and z components add to the total signal. In the plot, the total signal is multiplied by $\frac{1}{3}$ to enable the usage of the same color scale.

The N–O and N–H scattering signals in Fig. S23 do not show very strong differences in the Cartesian components.

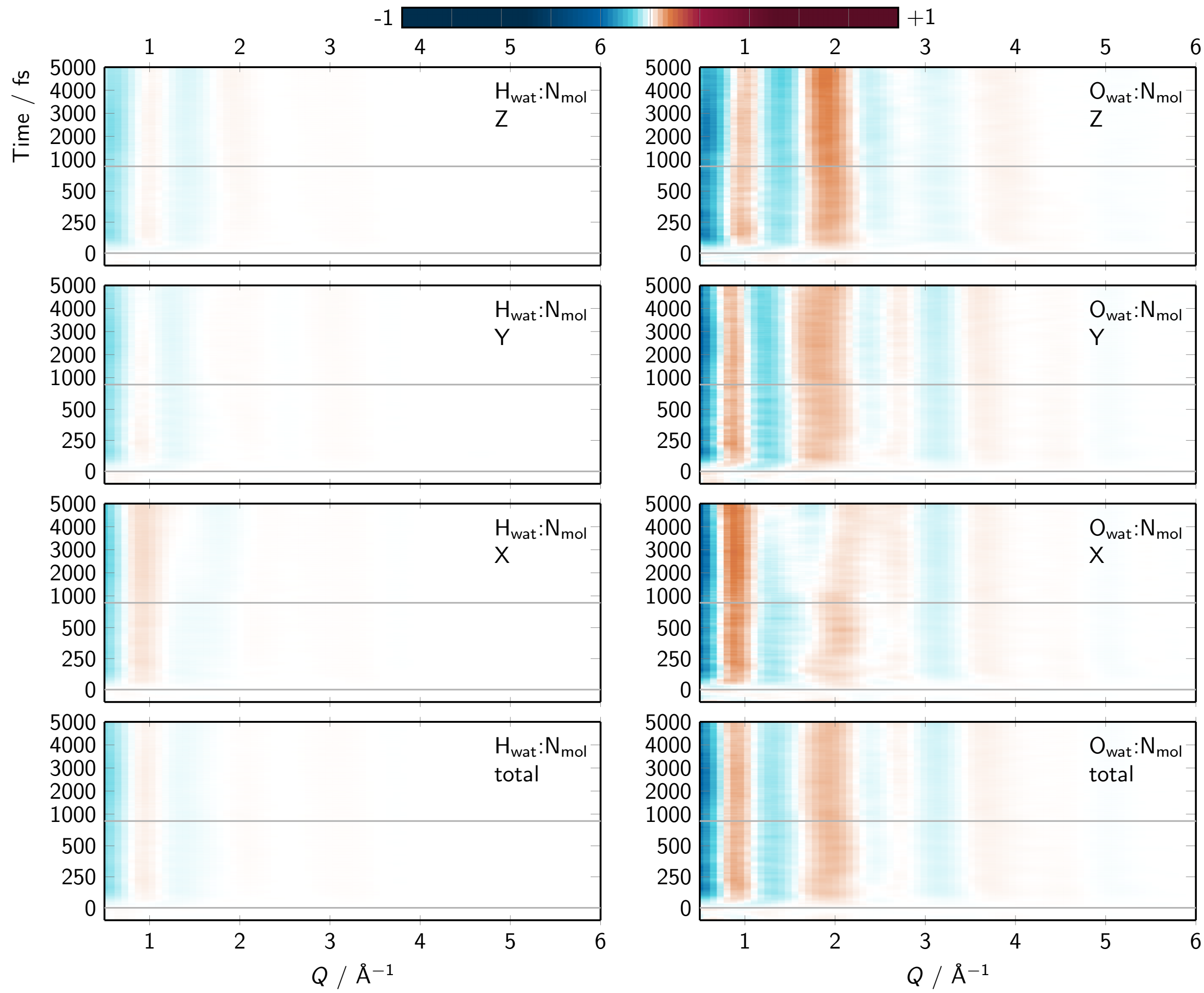

Figure S23: Time-resolved difference scattering due to N atoms of the molecule and H atoms (left) or O atoms (right) of the solvent. The bottom panels show the total difference scattering, whereas the panels above show the scattering due to the Cartesian components of the RDFs in x, y, and z direction (see labels). Note that the x, y, and z components add to the total signal. In the plot, the total signal is multiplied by $\frac{1}{3}$ to enable the usage of the same color scale.

In contrast, the C–O and C–H scattering signals in Fig. S24 differ dramatically between the Cartesian components. Around Q=1 Å^{-1}, the x component has a very large positive signal, presumably encoding the accumulation of water above and below the bipyridine ligand. In the y and z components, the scattering difference signals are significantly weaker and exhibit different intensity patterns.

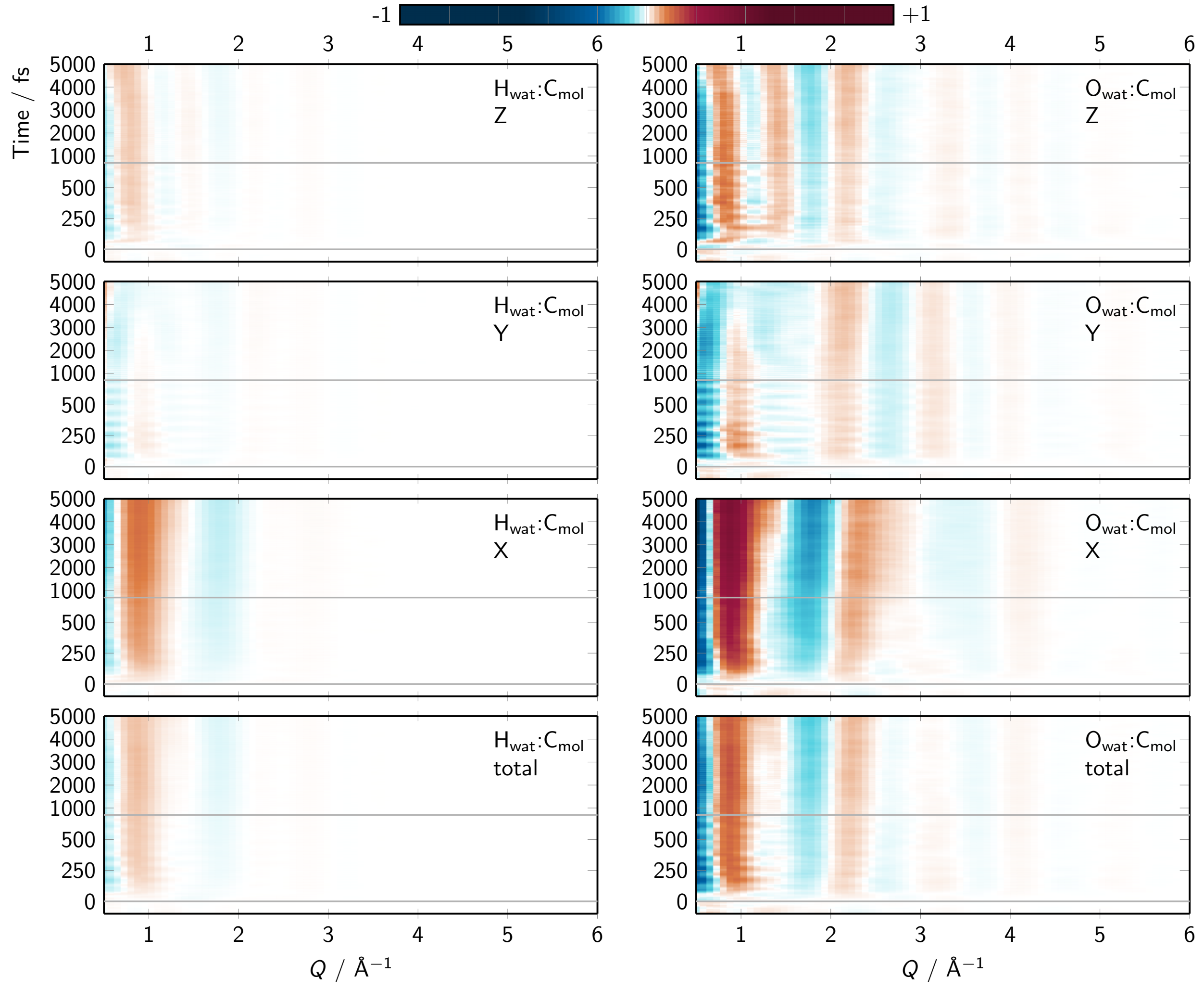

Figure S24: Time-resolved difference scattering due to C atoms of the molecule and H atoms (left) or O atoms (right) of the solvent. The bottom panels show the total difference scattering, whereas the panels above show the scattering due to the Cartesian components of the RDFs in x, y, and z direction (see labels). Note that the x, y, and z components add to the total signal. In the plot, the total signal is multiplied by $\frac{1}{3}$ to enable the usage of the same color scale.

As expected, the H–O and H–H scattering signals in Fig. S25 are very small and will not produce an observable signature.

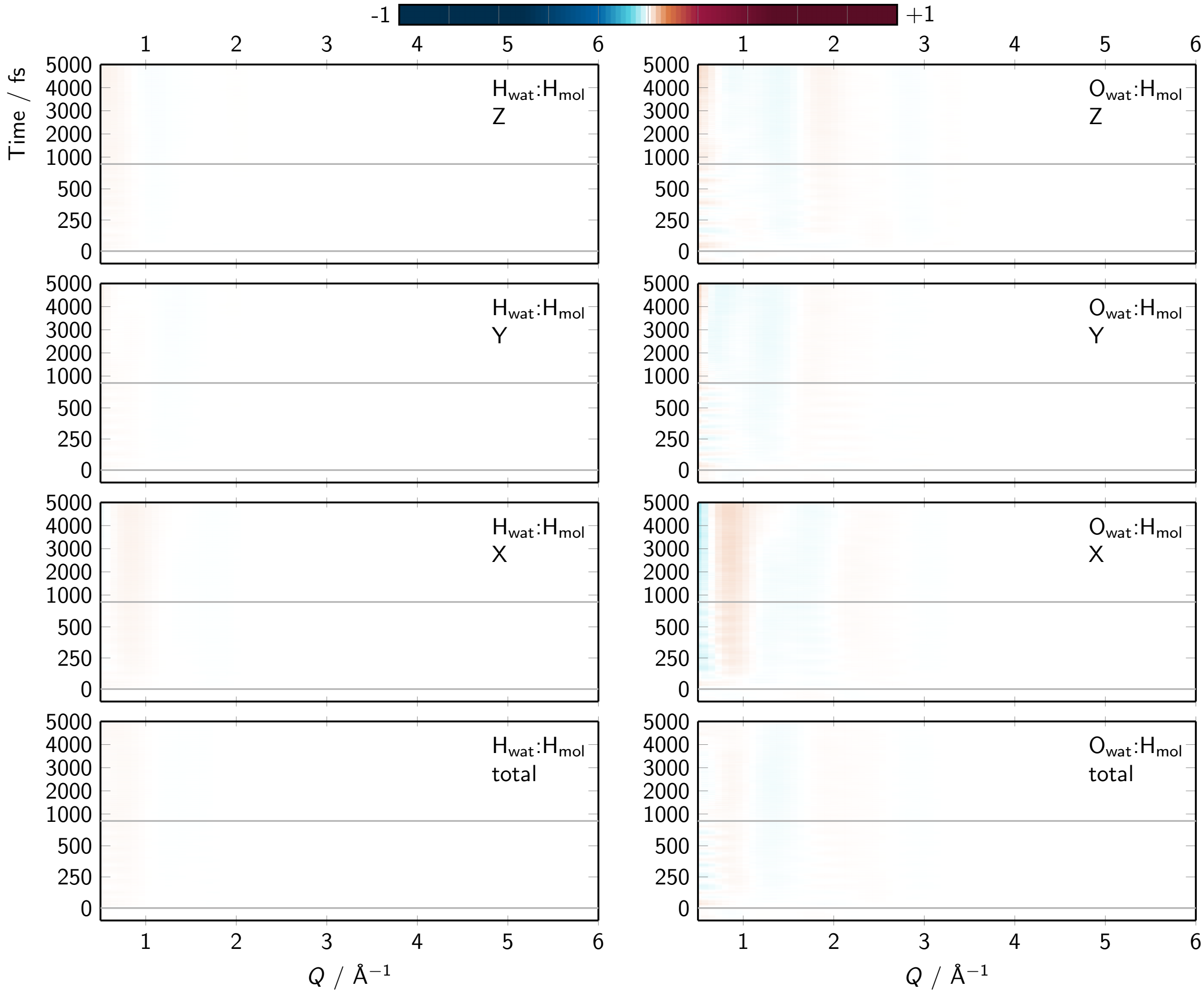

Figure S25: Time-resolved difference scattering due to H atoms of the molecule and H atoms (left) or O atoms (right) of the solvent. The bottom panels show the total difference scattering, whereas the panels above show the scattering due to the Cartesian components of the RDFs in x, y, and z direction (see labels). Note that the x, y, and z components add to the total signal. In the plot, the total signal is multiplied by $\frac{1}{3}$ to enable the usage of the same color scale.

The total scattering difference signal is shown in Fig. S26, exhibiting clear differences in the Cartesian components of the signal. Similar to the C–O scattering signal (Fig. S24), around Q=1 Å^{-1} a very large positive signal is seen, which only appears in the x component.

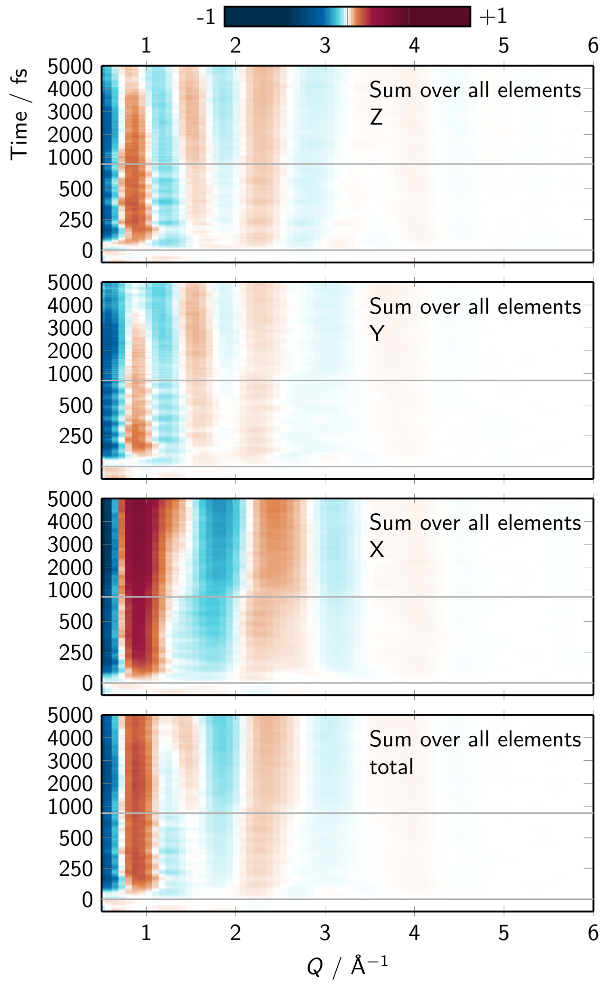

Figure S26: Sum of all time-resolved difference scattering signals. The bottom panels show the total difference scattering, whereas the panels above show the scattering due to the Cartesian components of the RDFs in x, y, and z direction (see labels). Note that the x, y, and z components add to the total signal. In the plot, the total signal is multiplied by $\frac{1}{3}$ to enable the usage of the same color scale.

We note that the scattering intensity depends on both the number of atoms and the atomic number. While Fe is the heaviest element in our system ($Z = 26$), there is only one Fe atom per molecule. In contrast, there are 14 C atoms ($Z = 6$), so overall the scattering due to C–O pairs is about three times as strong as the one due to Fe–O pairs. This explains why the directionality of the scattering due to the C–O pairs (Fig. S24) is relatively strong in the total scattering in Fig. S26. Hence, given that $[FeCN_4bpy]^{2+}$ can be sufficiently aligned experimentally, the strong scattering corresponding to the out-of-plane direction of the bipyridine ligand should allow the observation of the anisotropic solvent relaxation dynamics around $[FeCN_4bpy]^{2+}$.

References

(S1) Zederkof, D. B.; Møller, K. B.; Nielsen, M. M.; Haldrup, K.; González, L.; Mai, S. Resolving Femtosecond Solvent Reorganization Dynamics in an Iron Complex by Nonadiabatic Dynamics Simulations. *J. Am. Chem. Soc.* **2022**, *144*, 12861–12873.

(S2) Salomon, O.; Reiher, M.; Hess, B. A. Assertion and Validation of the Performance of the B3LYP⋆ Functional for the First Transition Metal Row and the G2 Test Set. *J. Chem. Phys.* **2002**, *117*, 4729–4737.

(S3) Frisch, M. J.; Trucks, G. W.; Schlegel, H. B.; Scuseria, G. E.; Robb, M. A.; Cheeseman, J. R.; Scalmani, G.; Barone, V.; Petersson, G. A.; Nakatsuji, H.; Li, X.; Caricato, M.; Marenich, A. V.; Bloino, J.; Janesko, B. G.; Gomperts, R.; Mennucci, B.; Hratchian, H. P.; Ortiz, J. V.; Izmaylov, A. F.; Sonnenberg, J. L.; Williams-Young, D.; Ding, F.; Lipparini, F.; Egidi, F.; Goings, J.; Peng, B.; Petrone, A.; Henderson, T.; Ranasinghe, D.; Zakrzewski, V. G.; Gao, J.; Rega, N.; Zheng, G.; Liang, W.; Hada, M.; Ehara, M.; Toyota, K.; Fukuda, R.; Hasegawa, J.; Ishida, M.; Nakajima, T.; Honda, Y.; Kitao, O.; Nakai, H.; Vreven, T.; Throssell, K.; Montgomery, J. A., Jr.; Peralta, J. E.; Ogliaro, F.; Bearpark, M. J.; Heyd, J. J.; Brothers, E. N.; Kudin, K. N.; Staroverov, V. N.; Keith, T. A.; Kobayashi, R.; Normand, J.; Raghavachari, K.; Rendell, A. P.; Burant, J. C.; Iyengar, S. S.; Tomasi, J.; Cossi, M.; Millam, J. M.; Klene, M.; Adamo, C.; Cammi, R.; Ochterski, J. W.; Martin, R. L.; Morokuma, K.; Farkas, O.; Foresman, J. B.; Fox, D. J. Gaussian 16 Revision C.01. 2016.

(S4) Proppe, J.; Gugler, S.; Reiher, M. Gaussian Process-Based Refinement of Dispersion Corrections. *J. Chem. Theory Comput.* **2019**, *15*, 6046–6060.

(S5) Neese, F. Software Update: The ORCA Program System—Version 5.0. *WIREs Comput. Mol. Sci.* **2022**, *12*, e1606.

(S6) Fumanal, M.; Plasser, F.; Mai, S.; Daniel, C.; Gindensperger, E. Interstate Vibronic Coupling Constants between Electronic Excited States for Complex Molecules. *J. Chem. Phys.* **2018**, *148*, 124119.

(S7) Plasser, F. TheoDORE: A Toolbox for a Detailed and Automated Analysis of Electronic Excited State Computations. *J. Chem. Phys.* **2020**, *152*, 084108.

(S8) Plasser, F.; Gómez, S.; Menger, M. F. S. J.; Mai, S.; González, L. Highly Efficient Surface Hopping Dynamics Using a Linear Vibronic Coupling Model. *Phys. Chem. Chem. Phys.* **2019**, *21*, 57–69.

(S9) Köppel, H.; Domcke, W.; Cederbaum, L. S. Theory of Vibronic Coupling in Linear Molecules. *J. Chem. Phys.* **1981**, *74*, 2945–2968.

(S10) Zobel, J. P.; Heindl, M.; Plasser, F.; Mai, S.; González, L. Surface Hopping Dynamics on Vibronic Coupling Models. *Acc. Chem. Res.* **2021**, *54*, 3760–3771.

(S11) Polonius, S.; Zhuravel, O.; Bachmair, B.; Mai, S. LVC/MM: A Hybrid Linear Vibronic Coupling/Molecular Mechanics Model with Distributed Multipole-Based Electrostatic Embedding for Highly Efficient Surface Hopping Dynamics in Solution. *J. Chem. Theory Comput.* **2023**, *19*, 7171–7186.

(S12) Abedi, M.; Levi, G.; Zederkof, D. B.; Henriksen, N. E.; Pápai, M.; Møller, K. B. Excited-State Solvation Structure of Transition Metal Complexes from Molecular Dynamics Simulations and Assessment of Partial Atomic Charge Methods. *Phys. Chem. Chem. Phys.* **2019**, *21*, 4082–4095.

(S13) Mai, S.; Gattuso, H.; Monari, A.; González, L. Novel Molecular-Dynamics-Based Protocols for Phase Space Sampling in Complex Systems. *Front. Chem.* **2018**, *6*, 495.

(S14) Case, D.; Aktulga, H.; Belfon, K.; Ben-Shalom, I.; Berryman, J.; Brozell, S.; Cerutti, D.; Cheatham III, T.; Cisneros, G.; Cruzeiro, V.; Darden, T.; Forouzesh, N.; Giambaşu, G.; Giese, T.; Gilson, M.; Gohlke, H.; Goetz, A.; Harris, J.; Izadi, S.; Izmailov, S.; Kasavajhala, K.; Kaymak, M.; King, E.; Kovalenko, A.; Kurtzman, T.; Lee, T.; Li, P.; Lin, C.; Liu, J.; Luchko, T.; Luo, R.; Machado, M.; Man, V.; Manathunga, M.; Merz, K.; Miao, Y.; Mikhailovskii, O.; Monard, G.; Nguyen, H.; O'Hearn, K.; Onufriev, A.; Pan, F.; Pantano, S.; Qi, R.; Rahnamoun, A.; Roe, D.; Roitberg, A.; Sagui, C.; Schott-Verdugo, S.; Shajan, A.; Shen, J.; Simmerling, C.; Skrynnikov, N.; Smith, J.; Swails, J.; Walker, R.; Wang, J.; Wang, J.; Wei, H.; Wu, X.; Wu, Y.; Xiong, Y.; Xue, Y.; York, D.; Zhao, S.; Zhu, Q.; Kollman, P. Amber 2023. 2023.

(S15) Wu, Y.; Tepper, H. L.; Voth, G. A. Flexible Simple Point-Charge Water Model with Improved Liquid-State Properties. *J. Chem. Phys.* **2006**, *124*, 024503.

(S16) Mai, S.; Avagliano, D.; Heindl, M.; Marquetand, P.; Menger, M. F. S. J.; Oppel, M.; Plasser, F.; Polonius, S.; Ruckenbauer, M.; Shu, Y.; Truhlar, D. G.; Zhang, L.; Zobel, P.; González, L. SHARC3.0: Surface Hopping Including Arbitrary Couplings — Program Package for Non-Adiabatic Dynamics. 2023.

(S17) Barbatti, M.; Lischka, H. Can the Nonadiabatic Photodynamics of Aminopyrimidine Be a Model for the Ultrafast Deactivation of Adenine? *J. Phys. Chem. A* **2007**, *111*, 2852–2858.

(S18) Mai, S.; Marquetand, P.; González, L. Nonadiabatic Dynamics: The SHARC Approach. *WIREs Comput. Mol. Sci.* **2018**, *8*, e1370.

(S19) Polonius, S.; Lehrner, D.; González, L.; Mai, S. Resolving Photoinduced Femtosecond Three-Dimensional Solute–Solvent Dynamics through Surface Hopping Simulations. *J. Chem. Theory Comput.* **2024**, *20*, 4738–4750.

(S20) Granucci, G.; Persico, M.; Toniolo, A. Direct Semiclassical Simulation of Photochemical Processes with Semiempirical Wave Functions. *J. Chem. Phys.* **2001**, *114*, 10608–10615.

(S21) Plasser, F.; Granucci, G.; Pittner, J.; Barbatti, M.; Persico, M.; Lischka, H. Surface Hopping Dynamics Using a Locally Diabatic Formalism: Charge Transfer in the

Ethylene Dimer Cation and Excited State Dynamics in the 2-Pyridone Dimer. *J. Chem. Phys.* **2012**, *137*, 22A514.

(S22) Granucci, G.; Persico, M.; Zoccante, A. Including Quantum Decoherence in Surface Hopping. *J. Chem. Phys.* **2010**, *133*, 134111.

(S23) Nangia, S.; Jasper, A. W.; Miller, T. F., III; Truhlar, D. G. Army Ants Algorithm for Rare Event Sampling of Delocalized Nonadiabatic Transitions by Trajectory Surface Hopping and the Estimation of Sampling Errors by the Bootstrap Method. *J. Chem. Phys.* **2004**, *120*, 3586–3597.

(S24) Kjær, K. S.; Kunnus, K.; Harlang, T. C. B.; Driel, T. B. V.; Ledbetter, K.; Hartsock, R. W.; Reinhard, M. E.; Koroidov, S.; Li, L.; Laursen, M. G.; Biasin, E.; Hansen, F. B.; Vester, P.; Christensen, M.; Haldrup, K.; Nielsen, M. M.; Chabera, P.; Liu, Y.; Tatsuno, H.; Timm, C.; Uhlig, J.; Sundstöm, V.; Németh, Z.; Szemes, D. S.; Bajnóczi, É.; Vankó, G.; Alonso-Mori, R.; Glownia, J. M.; Nelson, S.; Sikorski, M.; Sokaras, D.; Lemke, H. T.; Canton, S. E.; Wärnmark, K.; Persson, P.; Cordones, A. A.; Gaffney, K. J. Solvent Control of Charge Transfer Excited State Relaxation Pathways in $[Fe(2,2'\text{-Bipyridine})(CN)_4]^{2-}$. *Phys. Chem. Chem. Phys.* **2018**, *20*, 4238–4249.

(S25) Bayly, C. I.; Cieplak, P.; Cornell, W.; Kollman, P. A. A Well-Behaved Electrostatic Potential Based Method Using Charge Restraints for Deriving Atomic Charges: The RESP Model. *J. Phys. Chem.* **1993**, *97*, 10269–10280.

(S26) Dohn, A. O.; Biasin, E.; Haldrup, K.; Nielsen, M. M.; Henriksen, N. E.; Møller, K. B. On the Calculation of X-Ray Scattering Signals from Pairwise Radial Distribution Functions. *J. Phys. B: At. Mol. Opt. Phys.* **2015**, *48*, 244010.

(S27) Brown, P. J.; Fox, A. G.; Maslen, E. N.; O'Keefe, M. A.; Willis, B. T. M. *International Tables for Crystallography*; International Union of Crystallography, 2006; Vol. C; p 554–595, DOI: 10.1107/97809553602060000600.

(S28) Kim, J.; Kim, K. H.; Kim, J. G.; Kim, T. W.; Kim, Y.; Ihee, H. Anisotropic Picosecond X-ray Solution Scattering from Photoselectively Aligned Protein Molecules. *J. Phys. Chem. Lett.* **2011**, *2*, 350–356.

(S29) Brinkmann, L. U. L.; Hub, J. S. Anisotropic time-resolved solution X-ray scattering patterns from explicit-solvent molecular dynamics. *J. Chem. Phys.* **2015**, *143*, 104108.

(S30) Ross, A. E.; McCulloch, D. G.; McKenzie, D. R. Extending the Debye scattering equation for diffraction from a cylindrically averaged group of atoms: detecting molecular orientation at an interface. *Acta Crystallogr. A* **2020**, *76*, 468–473.

(S31) Zhang, Y.; Inouye, H.; Crowley, M.; Yu, L.; Kaeli, D.; Makowski, L. Diffraction pattern simulation of cellulose fibrils using distributed and quantized pair distances. *J. Appl. Cryst.* **2016**, *49*, 2244–2248.

(S32) Dearmond, M. K.; Fried, G. A. Langmuir-Blodgett Films of Transition Metal Complexes. 1996; http://dx.doi.org/10.1002/9780470166451.ch2.
